# Genome-scale metabolic reconstructions of *Bifidobacterium adolescentis* L2-32 and *Faecalibacterium prausnitzii* A2-165 and their interaction

**DOI:** 10.1186/1752-0509-8-41

**Published:** 2014-04-03

**Authors:** Ibrahim E El-Semman, Fredrik H Karlsson, Saeed Shoaie, Intawat Nookaew, Taysir H Soliman, Jens Nielsen

**Affiliations:** 1 Department of Chemical and Biological Engineering, Chalmers University of Technology, Gothenburg, Sweden; 2 Department of Mathematics, Faculty of Science, Assiut University, Assiut, Egypt; 3 Information Systems Department, Faculty of Computers and Information, Assiut University, Assiut, Egypt

**Keywords:** *Bifidobacterium adolescentis* L2-32, *Faecalibacterium prausnitzii* A2-165, Genome-scale metabolic model, Metabolic modeling of gut microbiota

## Abstract

**Background:**

The gut microbiota plays an important role in human health and disease by acting as a metabolic organ. Metagenomic sequencing has shown how dysbiosis in the gut microbiota is associated with human metabolic diseases such as obesity and diabetes. Modeling may assist to gain insight into the metabolic implication of an altered microbiota. Fast and accurate reconstruction of metabolic models for members of the gut microbiota, as well as methods to simulate a community of microorganisms, are therefore needed. The Integrated Microbial Genomes (IMG) database contains functional annotation for nearly 4,650 bacterial genomes. This tremendous new genomic information adds new opportunities for systems biology to reconstruct accurate genome scale metabolic models (GEMs).

**Results:**

Here we assembled a reaction data set containing 2,340 reactions obtained from existing genome-scale metabolic models, where each reaction is assigned with KEGG Orthology. The reaction data set was then used to reconstruct two genome scale metabolic models for gut microorganisms available in the IMG database *Bifidobacterium adolescentis* L2-32, which produces acetate during fermentation, and *Faecalibacterium prausnitzii* A2-165, which consumes acetate and produces butyrate. *F. prausnitzii* is less abundant in patients with Crohn’s disease and has been suggested to play an anti-inflammatory role in the gut ecosystem. The *B. adolescentis* model, iBif452, comprises 699 reactions and 611 unique metabolites. The *F. prausnitzii* model, iFap484, comprises 713 reactions and 621 unique metabolites. Each model was validated with *in vivo* data. We used OptCom and Flux Balance Analysis to simulate how both organisms interact.

**Conclusions:**

The consortium of iBif452 and iFap484 was applied to predict *F. prausnitzii’s* demand for acetate and production of butyrate which plays an essential role in colonic homeostasis and cancer prevention. The assembled reaction set is a useful tool to generate bacterial draft models from KEGG Orthology.

## Background

Metagenomic sequencing facilitates the study of a large number of microorganisms in environmental samples [1]. This technique has been used to study the composition of gut microbiota [2], its role in human metabolism [3,4] and its relation to diseases such as atherosclerosis [5], obesity [6,7] and Crohn’s disease [8]. In functional metagenomic studies, it is common to use KEGG Orthology (KO) [9] to annotate gene functions [10]. KO can be used to predict the composition ratio of microbial gene families and pathways from the human microbiome project [11]. The functional annotation for a large number of sequenced bacteria, nearly 4,650 bacterial genomes, is stored in the Integrated Microbial Genomes (IMG) database, and the genomes are mapped to KEGG pathway images [12]. This tremendous new genomic information adds a new opportunity for systems biology, as it enables use of information about genome content for prediction of metabolic phenotypes of species in the gut [13], or to develop community systems [14] or supra-model organisms [15]. Therefore, it is relevant to reconstruct accurate genome scale metabolic models (GEMs) from KO annotated by metagenomic analysis.

Several methods have been developed to reconstruct genome scale models from GEMs of closely related organisms [16], KEGG [16-18], and the Model SEED [19]. The RAVEN toolbox [16] has been used to generate GEMs for the eukaryotic microorganisms *Pichia stipitis* and *Pichia pastoris* using iIN800, a GEM of *Saccharomyces cerevisiae*[20]. However, this method requires a GEM of a closely related organism. The RAVEN toolbox has another function to solve this problem by assigning gene to KO using MUSCLE [21] and HMMER [22]. Then it generates the draft model by mapping KO to KEGG reactions. The web-based methods, FAME [17] and MicrobesFlux [18], are able to produce draft models for ~750 and ~1,200 KEGG genomes, respectively. The disadvantage of both FAME and MicrobesFlux is that they are limited to organisms already annotated in KEGG. The Model SEED can generate a draft model for a desired organism based on RAST annotation of genes [23]. Even though some of these methods have computational gap filling methods, there is still a need for manual curation to obtain a functional model. Manual curation is generally cumbersome and time-consuming. The lack of visualization, such as organization and readability of reactions and genes names into the model Excel file and KEGG maps, can hamper manual curation of generated draft models.

To facilitate generation of a draft model and manual gap filling, we assembled an organized reference reaction data set consisting of common microbial reactions, where every reaction is assigned with KOs. The reactions were collected from high quality GEMs and from Rhea, a manually annotated database of chemical reactions [24], but not from KEGG reactions. In spite of the accuracy of KEGG reactions, some reactions need to substantially manual curation for substrate and co-factor usage, and the reactions in reconstructed GEMs generally have to be well annotated in terms of substrate and co-factor usage and elemental balancing. Our reaction data set can, in principle, be used to generate draft models for all 4,650 bacteria in the IMG database, KEGG organisms, or other user-defined organisms annotated by KO.

Here, we used this reaction data set to generate high quality GEMs for two bacterial genomes from the IMG database: *B. adolescentis* L2-32 and *F. prausnitzii* A2-165. *Bifidobacterium* is a dominating genus in the phyla Actinobacteria present in the human gut microbiota and *Faecalibacterium* is the most abundant genus among the Firmicutes. Firmicutes, Bacteroidetes and Actinobacteria are the most highly abundant phyla in the human gut microbiota [2]. Both *Bifidobacterium* and *Faecalibacterium* interact with *Bacteroidetes*[25,26]. Moreover, *Bifidobacterium* produces acetate to protect the host from infection [27], and *Faecalibacterium* has a relation with Crohn disease [28]. Furthermore, the production of butyrate by *Faecalibacterium*, among others, has been associated with a healthy state [5,29,30].

Finally, we simulated and compared the interactions between the two organisms using two approaches for community modeling: Flux balance analysis [31-33] and OptCom [34].

## Methods

**Figure 1:**
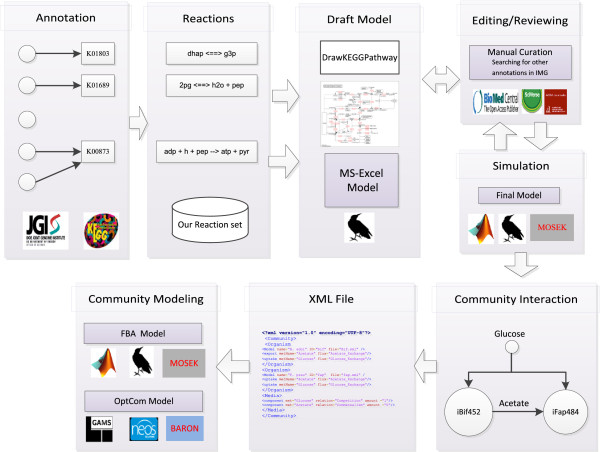
**Method summary. (I)** The gene assigned with KO for each studied organism was downloaded from the IMG database or KEGG. **(II)** The KO was mapped with the reaction data set. **(III)** A draft model was exported to MS-Excel format by our function *saveDraftModel*, the draft model was mapped to KEGG maps using our function *DrawPathway*. **(IV)** The draft was curated manually from literature and other gene annotations in IMG files such as TIGRFAMs and Pfam. After this, the model was simulated using RAVEN and MOSEK. **(V)** The community interaction design described how the organisms share growth medium components. **(VII)** Community interaction was converted to XML format. **(VIII)** Both optCom and FBA models were generated from XML files.

Figure 1 shows a summary of the methods developed and employed in this study. Reconstruction was based on the assignment of genes to KOs and this was used to generate a draft model from the reaction data set. A KEGG map viewer is helpful in identifying gaps in the reconstruction.

### Model reconstructions

#### Step 1 (Collecting reference reaction data set)

We assembled a reference reaction data set containing reactions assigned with KO for each KEGG map (see Additional file 1: Figure S1). For organisms Escherichia coli K-12 MG1655, Staphylococcus aureus N315 and Saccharomyces cerevisiae, we mapped the genes from each KEGG map to the corresponding reactions contained in the respective GEMs iAF1260 [35], iSB619 [36] and iTO977 [37]. The GEM reactions, together with the associated KOs, were then added to the reference reaction data set. In the case the organism had no genes for the enzyme or all GEMs lacked the reaction, we downloaded it from Rhea by the Matlab function *get_reaction_from_Rhea*. This function downloads Rhea reactions as XML format and prints them. To avoid the mismatched metabolite names between Rhea reactions and the reference reaction set, we evaluated the metabolite names in the Rhea reaction using its corresponding KEGG reaction. We retained Rhea metabolite names if they did not exist in the reference reaction data set, and the Rhea reaction and its corresponding KO were subsequently inserted into the reference reaction data set. iAF1260 reactions not present in any of the KEGG maps, especially transporters and reactions occurring in the cell wall, were inserted into the reference reaction data set with the corresponding KO as the corresponding gene. Moreover, we added the capsular polysaccharide and teichoic acid biosynthesis reactions, which are required for cell wall biosynthesis, from the GEM of *Lactobacillus plantarum* WCFS [38], because cell wall could be a significant fraction of gram dry weight of Gram-positive bacteria. Also we added the methane metabolism from the GEM of *Methanosarcina barkeri*[39] and added the siderophore group biosynthesis from the GEM of *Mycobacterium tuberculosis*[40]. Finally, the reactions were organized, ordered and made readable to facilitate the manual gap filling process.

#### Step2 (Generating draft models)

We downloaded the gene annotation for each studied organism from the IMG database [12]. We extracted the set *GK= (gene,KO)* for each organism from the downloaded IMG file using the function *get_gene_ko_from_img*. For organisms available in KEGG, the set *GK* can be obtained directly using the function *get_gene_ko_from_kegg_org_id,* otherwise the users can build the set *GK* themselves. The set *GK* was passed to the function *buildDraftModel* to extract reactions using KO identifiers from the reaction data set. The draft model was exported to an Excel file by the function *saveDraftModel*. Finally, we removed the exchange reaction for metabolites that were not participating in any cytosolic reaction. All the described functions are provided in Additional file 2 and can be used in the RAVEN toolbox.

#### Step 3 (Gap filling)

The gaps in each model were filled manually by mapping the model to KEGG maps and inserting the required reactions to ensure full connectivity in the model. To find genes for the filled reactions or metabolic genes, we extended the search to other available gene annotation in the IMG database. Both studied organisms have genes annotated by Pfam [41], TIGRFAMs [42], TC families [43] and METACYC [44]. Moreover*, B. adolescentis* L2-32 has gene annotations by SEED. Annotation with TC families has no specific gene assignment, so we ran a bidirectional blast between the TC protein sequence and each organism sequence. Also, we searched for the residual filled reactions by their enzyme name or EC number in the Pfam database to get the corresponding Pfam identifiers, and the corresponding genes were searched in IMG file. Additional file 3: Table S1 contains the results of this analysis.

### Flux balance analysis

Flux Balance Analysis (FBA) [45,46] was used to reconstruct and validate both models using the RAVEN toolbox and MOSEK (MOSEK Inc.) as a linear programming solver under the Matlab programming environment (Mathwork Inc.). Equation (1) describes the main formulation of FBA, where *S* is a stoichiometric matrix, *V* is a vector of flux values for all reactions and *C* is a weight vector for each flux in vector *V*. Typically all values in *C* are zero except the flux of the biomass reaction (*V*_*biomass*_) which is fixed to one. *UB* is the upper bound for the flux, and *LB* is the lower bound for the flux. In FBA, we assumed that the model grows in a growth medium (*GM*) which represents a set of metabolites *d*, such as a carbon source, ammonia, phosphate, sulfur. Certain exchange reactions, which carry flux from the medium to the model (*V*_*d*_), were fixed to the model uptake rate *g*_*d*_, for the metabolite *d.* For example 1 mmol/gDW/h of glucose uptake rate is fixed to flux (*V*_*glc*_) which carries glucose from the medium to the model.

(1) $$\begin{array}{llll} \text{Max} & Z & = & C^{T}V\\ S.t. & & & \\ & S.V & = & 0\\ & V & \leq & \text{UB}\\ & -V & \leq & -\text{LB}\\ & V_{d} & = & \text{gd},\;\forall d\;\in \;\text{GM} \end{array}$$

Both *B. adolescentis* L2-32 and *F. prausnitzii* A2-165 models grow anaerobically in rich media. We assumed that each model consumed ammonia as a source of nitrogen, phosphate, H_2_S or cysteine as a source of sulfur, nicotinate and all amino acids having transporter reactions. In addition, The *F. prausnitzii* A2-165 model consumed folic acid from the medium. Xanthine, uracil and urea transporters were closed.

The compositions of protein, RNA and DNA in the biomass were estimated from Neidhardt et al. [47]. The compositions of peptidoglycan and capsular polysaccharide are the same as in the GEM of *Lactobacillus plantarum* WCFS [38].

We used flux variability analysis [48] to evaluate the predicted fluxes by the COBRA toolbox function *fluxVariability*[49] (see Additional file 4: Table S2). To determine if a studied organism is able to grow on different carbon sources such as galactose, xylose or fructose, the transporter flux was fixed to 1 mmol/gDW/h and the biomass formation was optimized.

### Community modeling

In community studies each model was allowed to take up as much glucose as possible to maximize growth. In FBA simulations, *g*_*d*_ splits into two kinds of reactions with the first being a distribution of metabolite *d* to the different microorganisms in the community and the second being different transport reactions, where each reaction represents transport of glucose to one organism [31]. In OptCom, *g*_*d*_ became two variables: uptake variable for growth medium metabolite *d, uval*_*d*_^*k*^, or/and export variable for growth media, *eval*_*d*_^*k*^, where *k* = *bif or fap*  [34] and where bif is *B. adolescentis* L2-32 and fap is *F. prausnitzii* A2-165. Equation (2) gives the general OptCom problem formulation for this community. This problem has nonlinear constraint, so it cannot be solved using MOSEK. We therefore wrote a function *generateOptComModel* to convert RAVEN Matlab models to GAMS language (GAMS Development Corporation) and used the BARON solver hosted on the NEOS servers [50], a free optimization server, to solve the optimization problem.

(2) $$\begin{array}{lllllllll} \text{Max} & Z= & v_{\text{biomass}}^{\text{bif}} & + & \;v_{\text{biomass}}^{\text{fap}} & & & & \\ s.t. & & & & & & & & \\ & \text{Max} & Z^{\text{bif}} & = & v_{\text{biomass}}^{\text{bif}} & ,\text{Max} & Z^{\text{fap}} & = & v_{\text{biomass}}^{\text{fap}}\\ & s.t. & & & & s.t. & & & \\ & & S^{\text{bif}}V^{\text{bif}} & = & 0 & & S^{\text{fap}}V^{\text{fap}} & = & 0\\ & & V^{\text{bif}} & \leq & UB^{\text{bif}} & & V^{\text{fap}} & \leq & UB^{\text{fap}}\\ & & -V^{\text{bif}} & \leq & -LB^{\text{bif}} & & -V^{\text{fap}} & \leq & -LB^{\text{fap}}\\ & & v_{\text{glc}}^{\text{bif}} & \leq & \text{uva}l_{\text{glc}}^{\text{bif}} & & v_{\text{glc}}^{\text{fap}} & \leq & \text{uva}l_{\text{glc}}^{\text{fap}}\\ & & v_{\text{acetate}}^{\text{bif}} & = & \text{eva}l_{\text{acetate}}^{\text{bif}} & & v_{\text{acetate}}^{\text{fap}} & \leq & \text{uva}l_{\text{acetate}}^{\text{fap}}\\ \text{uva}l_{\text{glc}}^{\text{bif}} & + & \text{uva}l_{\text{glc}}^{\text{fap}} & = & \text{total}\_\text{glc} & & & & \\ \text{uva}l_{\text{acetate}}^{\text{fap}} & = & \text{eva}l_{\text{acetate}}^{\text{bif}} & & & & & & \end{array}$$

### Description of community using XML

We described a community structure without details of each model as XML format (see Additional file 5), because LibSBML fails to read an SBML containing a user defined attribute or XML tag for community features [51]. Both iBif452 and iFap484 competed for glucose while iFap484 consumed acetate produced by iBif452. The functions *generateOptComModel* and *generateComModel* used XML files to generate OptCom and FBA models.

## Results and discussion

### Reconstruction of reference reaction data set

GEMs elucidate how organisms consume nutrients, carbon source, ammonia, phosphate and autotrophic metabolites to build their biomass precursors and produce chemical byproducts [52]. The biochemical reactions included in a GEM are based on experimental or predicted function of enzymes contained by the studied organism [53]. Reconstructed GEMs share many components: exchange flux, transport, central metabolism, nucleotide, amino acids, cofactor biosynthesis, cell wall and lipid. Most GEMs use KEGG maps and literature to illustrate the content of each component, where it contains one or more KEGG maps. Additional file 1: Figure S1 (adapted from [54]) shows these components and the KEGG map name for each component and how flux distributes to each component and builds the necessary biomass precursors.

To cover the reactions in Additional file 1: Figure S1, we built a reference reaction data set from published GEMs and a manually curated reaction database Rhea. This reaction data set contained reactions for the central carbon metabolism (glycolysis, PP pathway, TCA, pyruvate), amino acids and nucleotide biosynthesis, cell wall (peptidoglycan, capsular polysaccharide and teichoic acid biosynthesis) and cofactors (folate, CoA and NAD+). We adopted the fatty acids biosynthesis and glycerophospholipd metabolism in iAF1260. Then we included reactions that connect other carbon resources, such as galactose and maltose to the main network.

The reactions were organized to facilitate manual revision and editing of newly reconstructed GEMs. Each reaction was assigned with KOs obtained from KEGG maps. The reference reaction data set comprises 2,340 reactions out of which 214 came from Rhea, 1256 unique metabolite and 2146 KOs (see Additional file 6: Table S3). The reference reaction data set was used to generate a draft model with an input file containing a gene and its KO. Additional file 7: Figure S2 shows how the reaction set covers KEGG maps.

### GEMs description

Table 1 shows statistics for the reconstructed GEMs and comparison with draft models generated by Model SEED. The iBif452 model comprises 699 reactions, 611 unique metabolites and 452 genes constituting 18.62% of the total number of genes. It contains 6 genes from TIGRFAMs and one gene from Pfam. It needed 9 reactions to become a connected model. Model iFap484 comprises 713 reactions, 621 unique metabolites and 484 genes, constituting 13.93% of the total number of genes. It contains 6 genes from TIGRFAMs and two genes from Pfam. It needed 16 reactions to become a connected model. Additional file 8 contains both models as Excel and SBML format.

**Table 1:** **Description of GEMS: iBif452 and iFap484 of *B. adolescentis *L2-32 and *F. prausnitzii *A2-165 in comparison with draft model generating by Model SEED**

	***B. adolescentis *L2-32**		***F. prausnitzii *A2-165**	
	**iBif452**	**SEED draft**	**iFap484**	**Seed draft**
Reactions	699	663	713	787
Unique metabolite	611	691	621	798
Genes	452	543	484	586
Reactions without genes	84		90	
Exchange	59		60	
Transporter	12		10	
Spontaneous	4		4	
FILL	9		16	
Genes from other annotations	7		8	
Coding genes	2428		3475	

Both iBif452 and iFap484 have reactions for the central carbon metabolism and can utilize other sole carbon sources than glucose, as reported in *in vivo* studies. iBif452 can utilize galactose, fructose and maltose, which is consistent with *in vivo* studies [55]. The iFap484 can also utilize galactose and maltose but cannot utilize xylose, which is also consistent with *in vivo* studies [56]. iBif452 features the bifid shunt pathway or the F6PPK pathway representing a special *Bifidobacteria* pathway converting glucose to pyruvate (see Additional file 9: Figure S3) [57-59]. The F6PPK pathway includes fructose-6-phosphate phosphoketolase converting D-Fructose 6-phosphate to Acetyl phosphate and D-Erythrose 4-phosphate, compared to the common part of glycolysis with 6-phosphofructokinase and fructose-1,6-bisphosphate aldolase. iFap484 has a *Faecalibacterium prausnitzii* butyrate producing pathway (see Additional file 10: Figure S4) [60]. Neither iBif452 nor iFap484 produces anything when the glucose uptake rate is 0 mmol/gDW/h and the objective function is biomass or ATP non-growth association maintenance, so the models did not generate energy or matter from nothing. In spite of the two models were validated with FBA in the following two sections, the comprehensive validation of the GEMs needs extensive experiments. Since these bacteria are not yet well-studied, we think the two models based on recently sequenced, may assist a lot to overcome some important questions, computing phenotypic states and describing the genotype-phenotype relationships.

### *Bifidobacterium adolescentis adolescentis* L2-32 validation

*Bifidobacterium* has predicted genes for biosynthesis of all 20 amino acids, purines and pyrimidines [57]. However, *Bifidobacterium* only grows in complex media, probably because some of the genes in the biosynthetic pathway for amino acids are non-functional [57]. We assumed that iBif452 grows in media containing 12 amino acids for which it has transporter reactions.

The iBif452 model did not produce lactate when biomass or ATP production was optimized, while *Bifidobacterium* produces acetate, lactate, formate and ethanol *in vivo*. Under glucose limitation, *Bifidobacterium* does not produce lactate because it tries to maximize energy production by cleaving pyruvate to acetyl phosphate and formate [61]. Furthermore, the specific rate of sugar consumption affects the amount of lactate production. For example, the organism produces a large amount of lactate when it has a rapid sugar consumption, but produces a small amount of lactate when it consumes a less preferred sugar like oligofructose [62].

To study the ability of the model iBif452 to produce lactate, we maximized ATP production for non-growth association maintenance, i.e., the reaction ATP + H2O = > ADP + Phosphate + H. The model produced 3 mmol of ATP per 1 mmol of glucose and produced only acetate, formate and ethanol. When the model was constrained to produce 1 mmol of lactate per 1 mmol of glucose, it produced 2.5 mmol of ATP and 1.5 mmol of acetate per 1 mmol of glucose.

**Figure 2:**
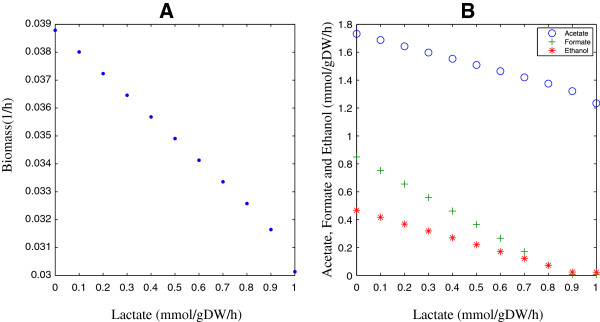
**The effect of lactate production on the iBif452 Model. (A)** The biomass decreases with increasing the lactate production. **(B)** The production of acetate, formate, and ethanol decrease with increasing the lactate production.

Figure 2 shows the effect of lactate production on the iBif452 model when the biomass is an objective function. In Figure 2A, the model achieved maximum biomass when there was no production of lactate. In Figure 2B, with increased production of lactate, the production rate of acetate, ethanol and formate decreased. The model was still able to produce acetate without formate because *Bifidobacterium* has the fructose-6-phosphate phosphoketolase enzyme, which converts fructose 6-phosphate into erythrose 4-phosphate and acetyl phosphate and the latter can yield an ATP when metabolized to acetate. Flux variability analysis showed the differences between maximum and minimum fluxes for acetate, formate and ethanol were 0.0003, 0.011 and 0.011 mmol per 1 mmol of glucose.

The last results showed that the model aimed to generate ATP by converting pyruvate to acetate through acetyl-CoA and acetyl-phosphate and it therefore has to regenerate NAD + by forming ethanol, as this is only way this co-factor can be balanced when there is formation of acetate (See Additional file 9: Figure S3). Although the model predicts a flux distribution for the theoretical ratio between acetate and lactate in *Bifidobacterium*, it fails to predict the amount of lactate just like previous GEMs of lactic acid bacteria [63]. To overcome this problem, Oliveira et al. constrained the pyruvate formate lyase reaction to an interval to deal with lactate production in a GEM of *L. Lactic*[64]. Bas Teusink et al. fixed the measured flux in the GEM of *L. plantarum* WCFS1 [38]. Milan et al. added new enzyme turnover parameter to avoid metabolism overflow in GEM of *L.lactis*[65] based on flux balance analysis with molecular crowding [66]. Finally, Bas Teusink et al. showed that *L. plantarum* optimizes its yield when it grows with glycerol to support the prediction of GEM in lactic acid bacteria [67].

In the present work, we constrained the lactate flux in the model. When we constrain lactate production with a yield of 1 (mmol/mmol of glucose), the model produces acetate with a yield of 1.23 (mmol/mmol glucose), as listed in Table 2. Flux variability analysis shows acetate yield having a 0.007 (mmol/mmol glucose) difference between maximum and minimum fluxes. When the ATP production for non-growth association maintenance was used as an objective function, it produced acetate with a yield of 1.5 (mmol/mmol glucose). Flux variability analysis showed that the acetate yield was not different between the maximum and minimum fluxes. Finally, when the model was constrained to produce 0.21 mol of lactate per mol of glucose, it had a growth yield of 37.2 gDW per mol of glucose, which was very close to the *in vivo. Bifidobacterium* growth yield of 37.4 gDW per mol of glucose [68].

**Table 2:** **Comparison between *in-silco *prediction of short chain fatty acids of iBif452 and iFap484 with experimental data**

		**Yield (mmol / mmol glucose)**			
		**Formate**	**Acetate**	**Lactate**	**Butyrate**
iBif452					
	Experimental [62]		1.47	1	
	Prediction (Biomass)		1.23	1	
	Prediction (ATP)		1.5	1	
iFap484					
	Experimental [60]	0.97 ± 0.12	−0.96 ± 0.12	0.05 ± 0.01	1.045 ± 0.15
	Prediction	1.77	−1.39	0	1.62

### *Faecalibacterium prausnitzii* A2-165 validation

To study the effect of external acetate on butyrate production in *Faecalibacterium prausnitzii* A2-165, biomass production was used as an objective function. The model produces butyrate with a yield of 1.62 (mmol butyrate/mmol glucose) and co-consumes 1.39 mmol of acetate per mmol of glucose. The ratio of acetate uptake to butyrate production was 85.8%, which is close to the 85-90% observed in *in vivo* studies of *F. prausnitzii*[69]. Flux variability analysis shows that the acetate, butyrate, and formate have a difference of 0.0005, 0.014, and 0.014 mmol/gDW/h between maximum and minimum fluxes, respectively. Table 2 shows the comparison between these values with *in vivo* studies [60], where *F. prausnitzii* consumed 10 mM of glucose and 9.55 ± 1.2 mM of acetate to produce 10.45 ± 1.53 mM of butyrate.

**Figure 3:**
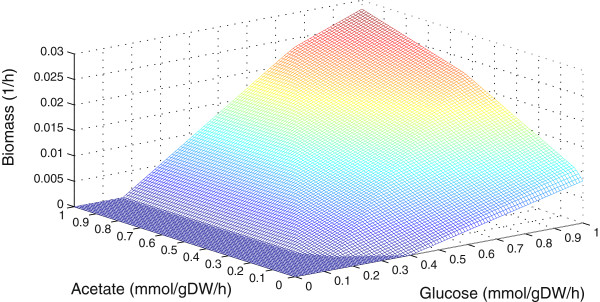
**The effect of glucose and acetate on the growth of iFap484.** Glucose and acetate uptake rate varied between 0 to 1 mmol/gDW/h.

Figure 3 shows a sensitivity analysis of the effect of acetate and glucose on iFap484 with biomass as an objective function. The acetate and glucose uptake rate varied from 0 to 1 mmol/gDW/h. iFap484 cannot grow without glucose and grows poorly without acetate. This result is similar to *in vivo* studies of *F. prausnitzii* A2-165, that shows poor growth without acetate in the medium [69] and the *F. prausnitzii* strains L2-6 and ATCC 27766 cannot grow without acetate [70]. The model predicted increase in growth rate with acetate supplied was 3.93-fold, close to the *in vivo* value, where the increase in growth rate is 3.6-fold [60].

### Community simulation

Both OptCom and FBA methods were applied to iBif452 and iFap484 to simulate how *B. adolescentis* L2-32 and *F. prausnitzii* A2-165 co-culture together. Both organisms compete for 1 mmol/gDW/h of glucose to maximize their growth. The model iBif452 generates acetate and iFap484 consumes acetate to produce butyrate, which plays a critical role in colonic homeostasis and cancer prevention [71-73].

**Figure 4:**
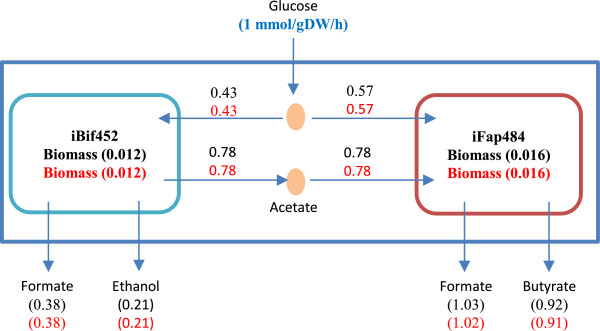
**Simulation summary results using OptCom and FBA methods when iBif452 and iFap484 grow together on glucose.** The black numbers are fluxes predicted by OptCom, the red numbers are fluxes predicted by the FBA method. ATP non-growth association maintenance was fixed at 0.4 and 0.5 mmol/gDW/h in the iFap484 and iBif452 models respectively. The unit of ATP, acetate, lactate, ethanol, formate and butyrate is mmol/gDW/h. The unit of biomass is (1/h).

Figure 4 depicts the prediction of fluxes calculated using both methods: OptCom and FBA, where biomass was an objective function. iFap484 consumed 0.57 mmol/gDW/h of glucose and all the acetate produced by iBif452 to produce 0.92 mmol/gDW/h of butyrate. With flux variability analysis, the differences between maximum and minimum yield of acetate and butyrate were 0.00004 and 0.007 (mmol/mmol of glucose) respectively. There was no significant difference between OptCom and FBA simulations. However, for this simple system, FBA provided faster simulation than OptCom.

**Figure 5:**
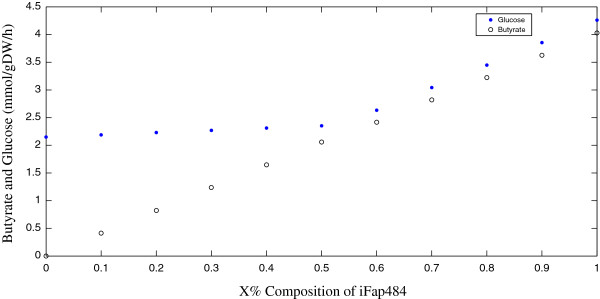
**Abundances analysis of iFap484 and iBif452.** How the amount of butyrate and the total glucose consumption change with different compositions of iFap484 and iBif452.

Finally, FBA was used to simulate how a consortium of iBif452 and iFap484 interact. The composition of the consortia was varied from 0% to 100% iFap484, while keeping the total biomass constant, and the objective function was minimization of glucose uptake rate in both iBif452 and iFap484. The biomass production rate was fixed at × % in iFap484 and (1-×) % in iBif452 of total biomass, where the total biomass growth rate was 0.1 (1/h). Figure 5 shows the increase in butyrate production as iFap484 composition increases.

## Conclusion

We assembled a reaction set from published GEMs, where each reaction is assigned with KO. This reaction set was used to generate draft GEMs for each non-KEGG organisms. It represented a simple method to generate bacterial draft models from KEGG KO, instead of generating it from KGML [74]. The description of a community as a XML format can be used together with the two community simulation methods Optcom and FBA. This saves time and effort when performing community modeling.

Community simulations of an acetate producer *B. adolescentis adolescentis* L2-32 and an acetate consumer *F. prausnitzii* A2-165 provided insights into metabolic cross talk between these two members of the gut microbiota. It shows the importance of acetate supply to butyrate production, since the growth and production of *Faecalibacterium prausnitzii* is severely hampered by limited acetate supply. This is an initial attempt to approach the very complex ecosystem and metabolic organ that the gut microbiota constitutes.

## Competing interests

The authors declare that they have no competing interests.

## Authors’ contributions

IEE-L developed the methods, reconstructed the two genome-scale metabolic models, performed the simulations and drafted the manuscript. FK and SS assisted in the process of model reconstruction and edited the manuscript. IN, THS and JN supervised the work and edited the manuscript. JN conceived and designed the project. All authors read and approved the final manuscript.

## Supplementary Material

Additional file 1: Figure S1 Mapping the main component of GEM to KEGG pathway maps (adapted from [54]). Click here for file

Additional file 2 Matlab funtions used in the paper. Click here for file

Additional file 3: Table S1 Genes with annotations: TIGRFAMs, KOs, SEED, TC number. Click here for file

Additional file 4: Table S2 Flux variability analysis for prediction fluxes in the study. Click here for file

Additional file 5 The structure of community as XML format. Click here for file

Additional file 6: Table S3 Reference reaction data set. Click here for file

Additional file 7: Figure S2 Mapping the assembled reaction set to KEGG metabolic pathways maps. Click here for file

Additional file 8 **The two models in SBML and MS Excel format.** The two models are also available through the Human Metabolic Atlass http://www.metabolicatlas.com/downloads/micro. Click here for file

Additional file 9: Figure S3 Main carbon metabolism in *Bifidobacterium adolescentis adolescentis* L2-32. Click here for file

Additional file 10: Figure S4 Main carbon metabolism in *Faecalibacterium prausnitzii* A2-165. Click here for file
