# Supplementary material for: Genome-scale metabolic reconstructions of Bifidobacterium adolescentis L2-32 and Faecalibacterium prausnitzii A2-165 and their interaction
Source: BMC Syst Biol. 2014 Apr 3;8:41. doi: 10.1186/1752-0509-8-41 (PMC4108055; doi:10.1186/1752-0509-8-41)
Supplement: Additional file 1: Figure S1 — Mapping the main component of GEM to KEGG pathway maps (adapted from [54]). [file 1752-0509-8-41-S1.pdf]

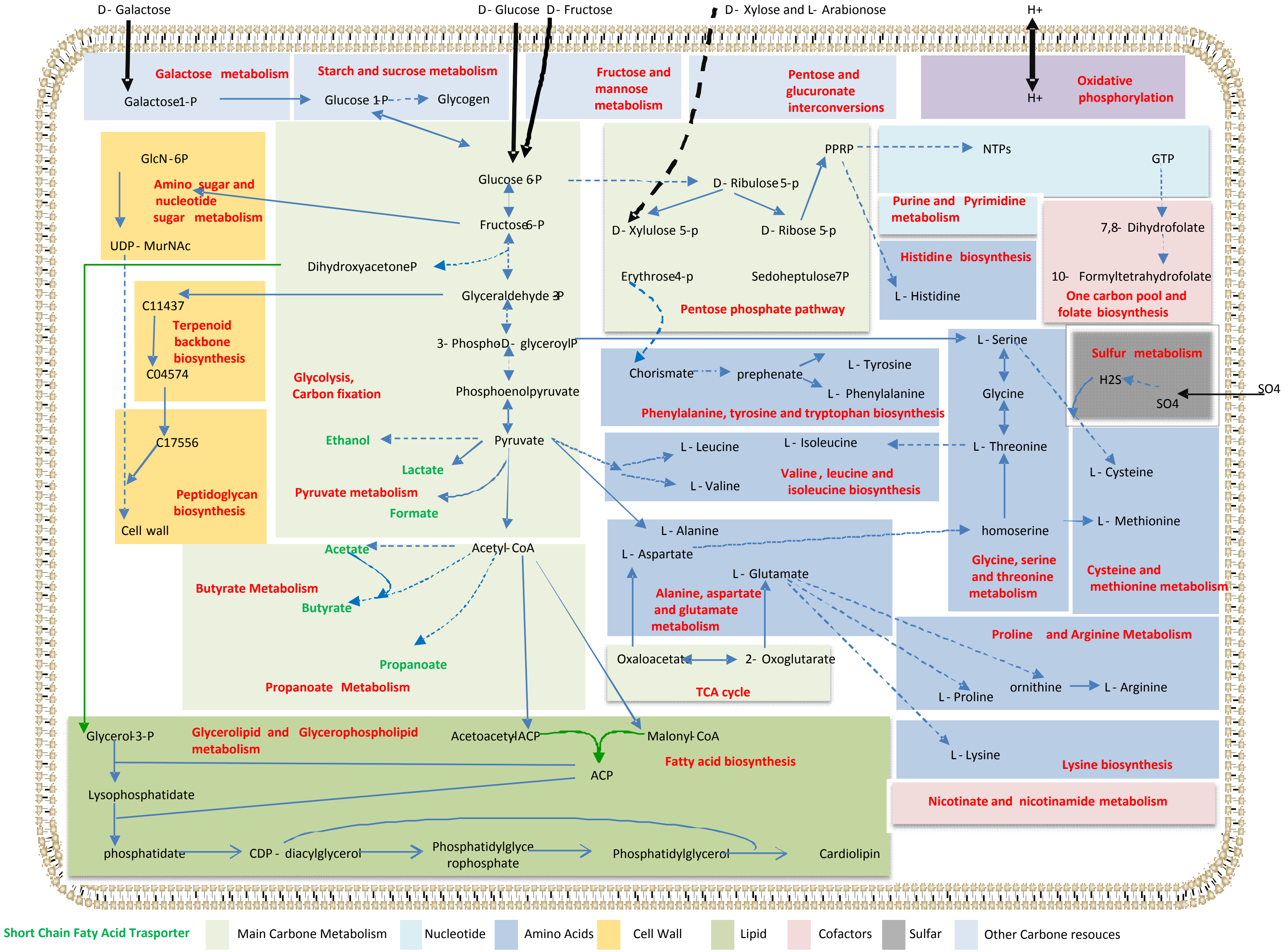
D- Galactose
D- Glucose D- Fructose
D- Xylose and L- Arabionose
H+
Galactose metabolism
Galactose1-P
Starch and sucrose metabolism
Glucose 1-P
Glycogen
Fructose and mannose metabolism
Pentose and glucuronate interconversions
Oxidative phosphorylation
H+
GlcN-6P
Amino sugar and nucleotide sugar metabolism
UDP- MurNAc
Glucose 6-P
Fructose6-P
DihydroxyacetoneP
Glyceraldehyde 3P
3- PhosphoD- glyceroylP
Phosphoenolpyruvate
Pyruvate
Glycolysis, Carbon fixation
Ethanol
Lactate
Pyruvate metabolism
Formate
C11437
Terpenoid backbone biosynthesis
C04574
C17556
Peptidoglycan biosynthesis
Cell wall
PPRP
D- Ribulose 5-p
D- Xylulose 5-p
D- Ribose 5-p
Erythrose4-p
Sedoheptulose7P
Pentose phosphate pathway
NTPs
GTP
Purine and Pyrimidine metabolism
Histidine biosynthesis
L- Histidine
7,8- Dihydrofolate
10- Formyltetrahydrofolate
One carbon pool and folate biosynthesis
L- Serine
Glycine
L- Threonine
homoserine
Glycine, serine and threonine metabolism
Sulfur metabolism
H2S
SO4
SO4
L- Cysteine
L- Methionine
Cysteine and methionine metabolism
Chorismate
prephenate
L- Tyrosine
L- Phenylalanine
Phenylalanine, tyrosine and tryptophan biosynthesis
L- Leucine
L- Isoleucine
L- Valine
Valine, leucine and isoleucine biosynthesis
L- Alanine
L- Aspartate
L- Glutamate
Alanine, aspartate and glutamate metabolism
Oxaloacetate
2- Oxoglutarate
TCA cycle
Proline and Arginine Metabolism
L- Proline
ornithine
L- Arginine
L- Lysine
Lysine biosynthesis
Nicotinate and nicotinamide metabolism
Acetyl- CoA
Acetate
Butyrate Metabolism
Butyrate
Propanoate
Propanoate Metabolism
Glycerol-3-P
Glycerolipid and Glycerophospholipid metabolism
AcetoacetylACP
Malonyl- CoA
Fatty acid biosynthesis
ACP
Lysophosphatidate
phosphatidate
CDP- diacylglycerol
Phosphatidylglycerophosphate
Phosphatidylglycerol
Cardiolipin
Short Chain Faty Acid Trasporter
Main Carbone Metabolism
Nucleotide
Amino Acids
Cell Wall
Lipid
Cofactors
Sulfar
Other Carbone resouces
