# Supplementary material for: Genome-scale metabolic reconstructions of Bifidobacterium adolescentis L2-32 and Faecalibacterium prausnitzii A2-165 and their interaction
Source: BMC Syst Biol. 2014 Apr 3;8:41. doi: 10.1186/1752-0509-8-41 (PMC4108055; doi:10.1186/1752-0509-8-41)
Supplement: Additional file 7: Figure S2 — Mapping the assembled reaction set to KEGG metabolic pathways maps. [file 1752-0509-8-41-S7.pdf]

CARBON METABOLISM

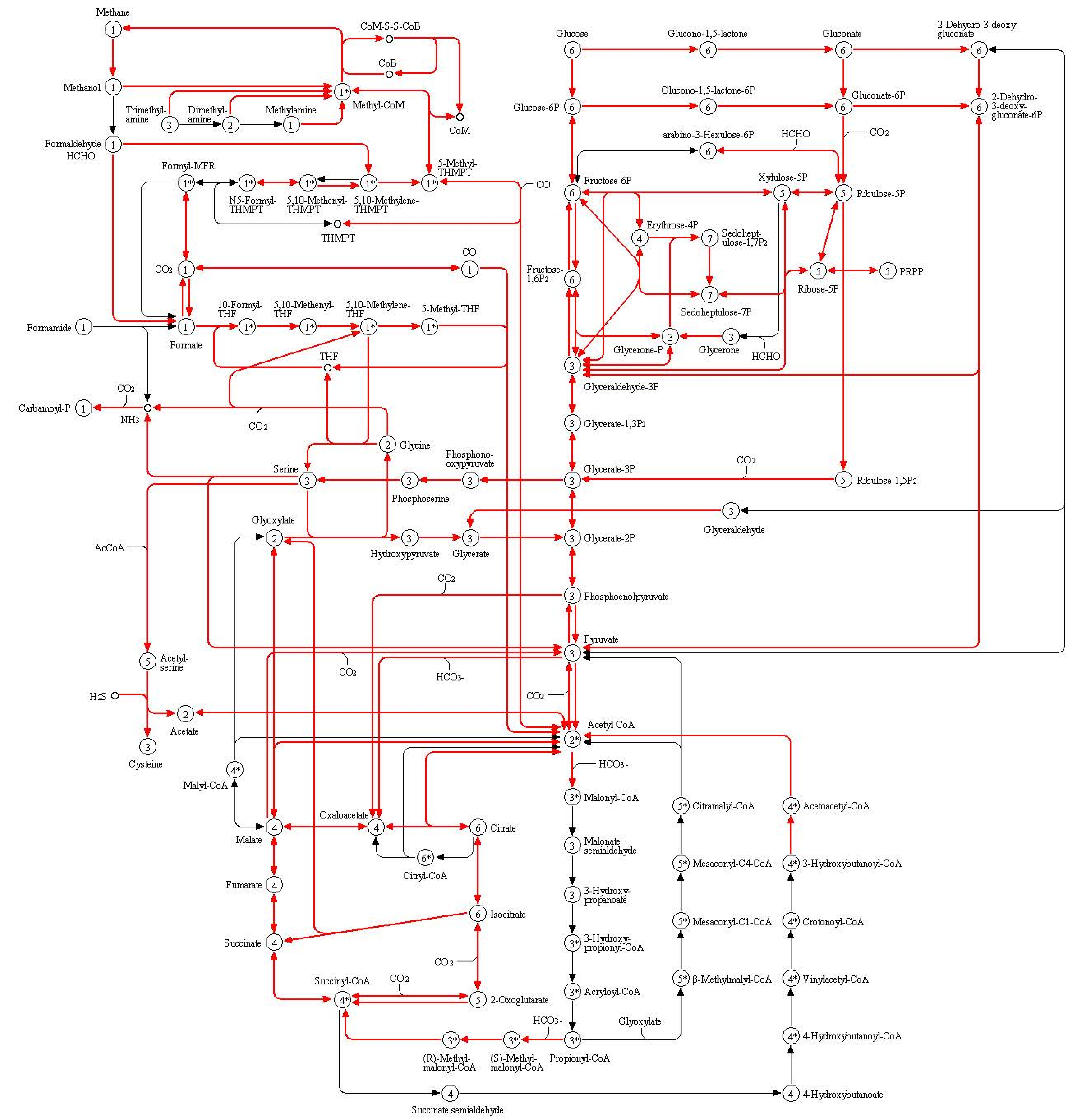

01200 8/26/13
(c) Kanehisa Laboratories

BIOSYNTHESIS OF AMINO ACIDS

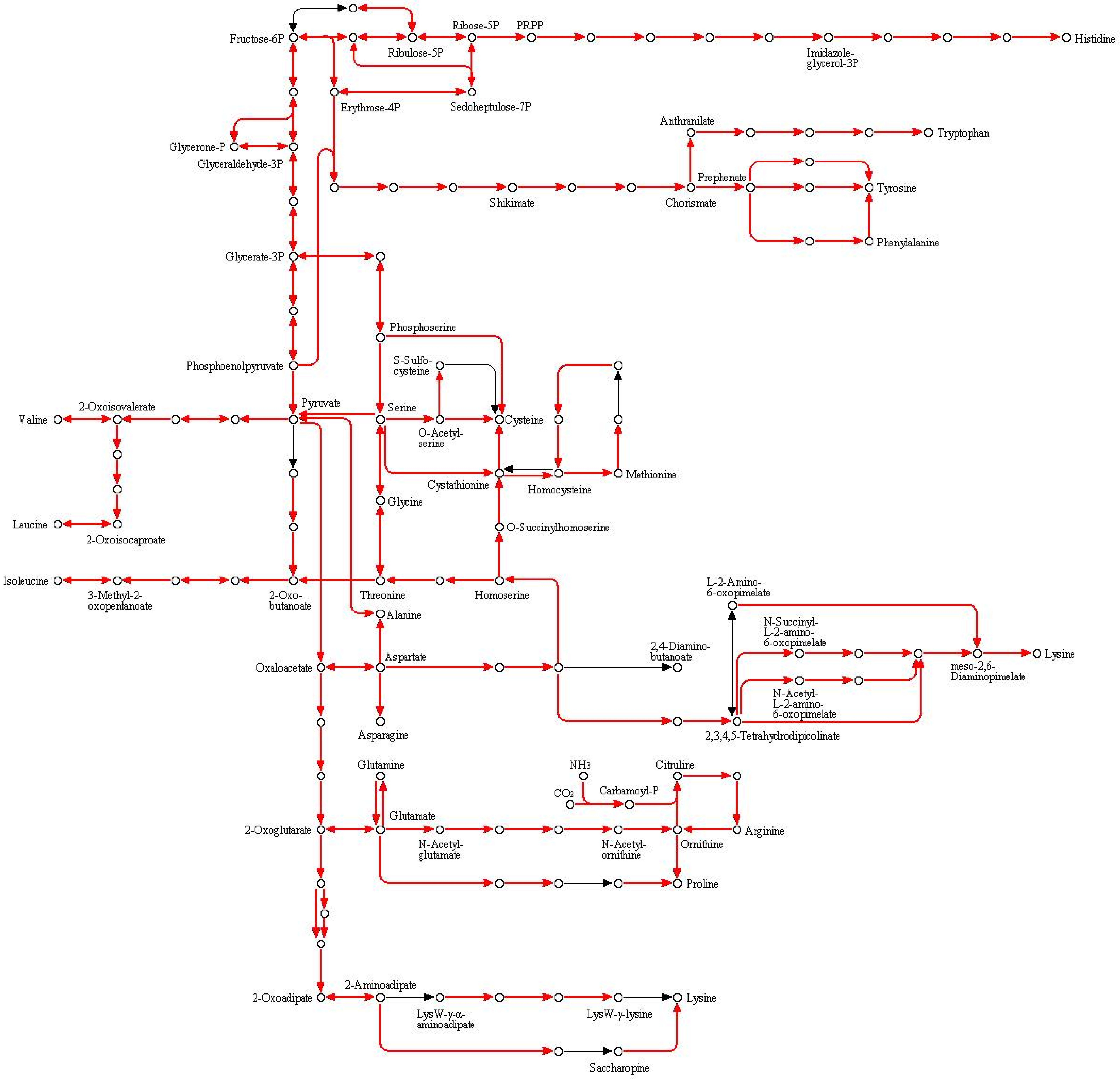

01230 9/3/13

(c) Kanehisa Laboratories

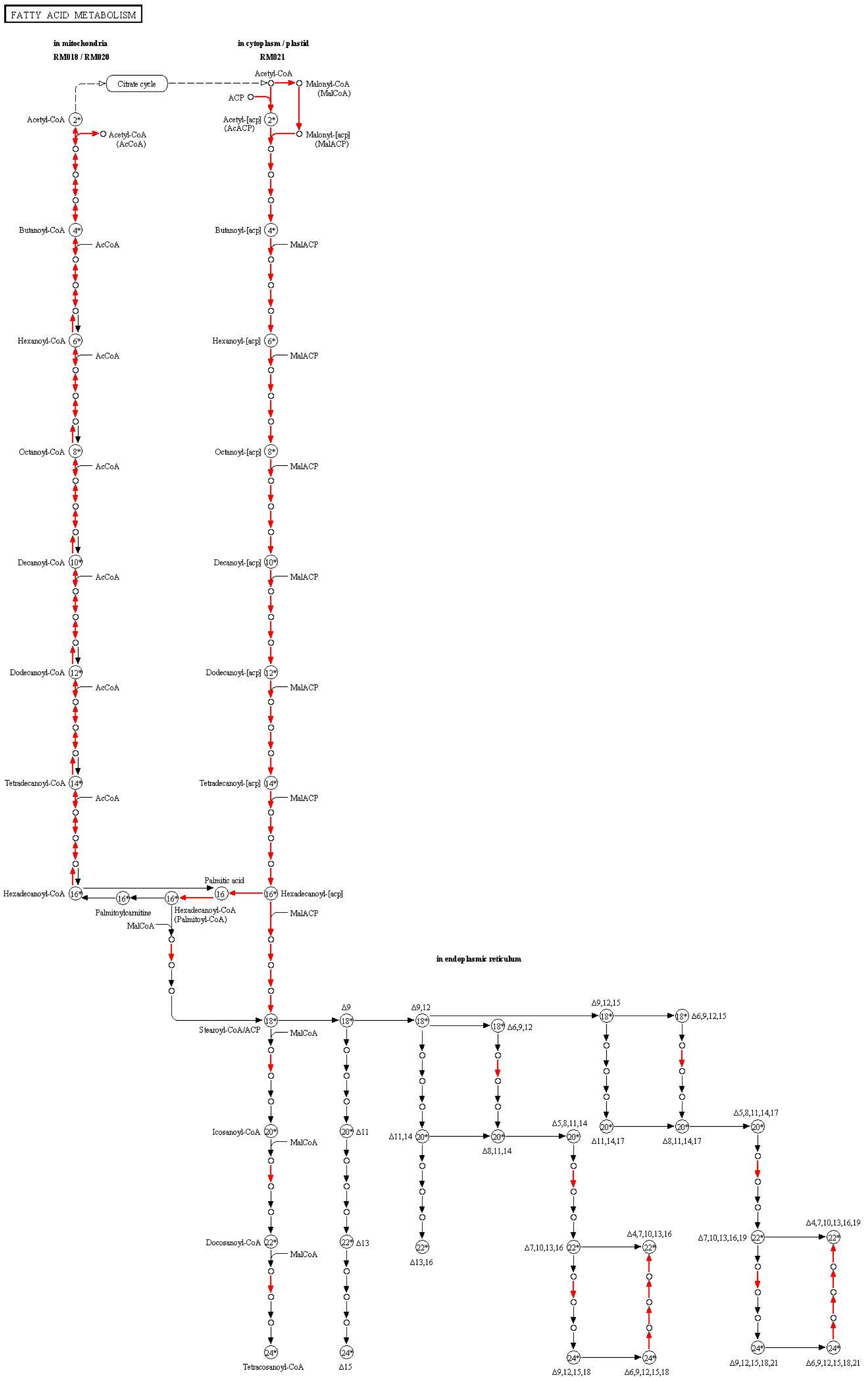

01212 10/8/13
(c) Kanehisa Laboratories

PURINE METABOLISM

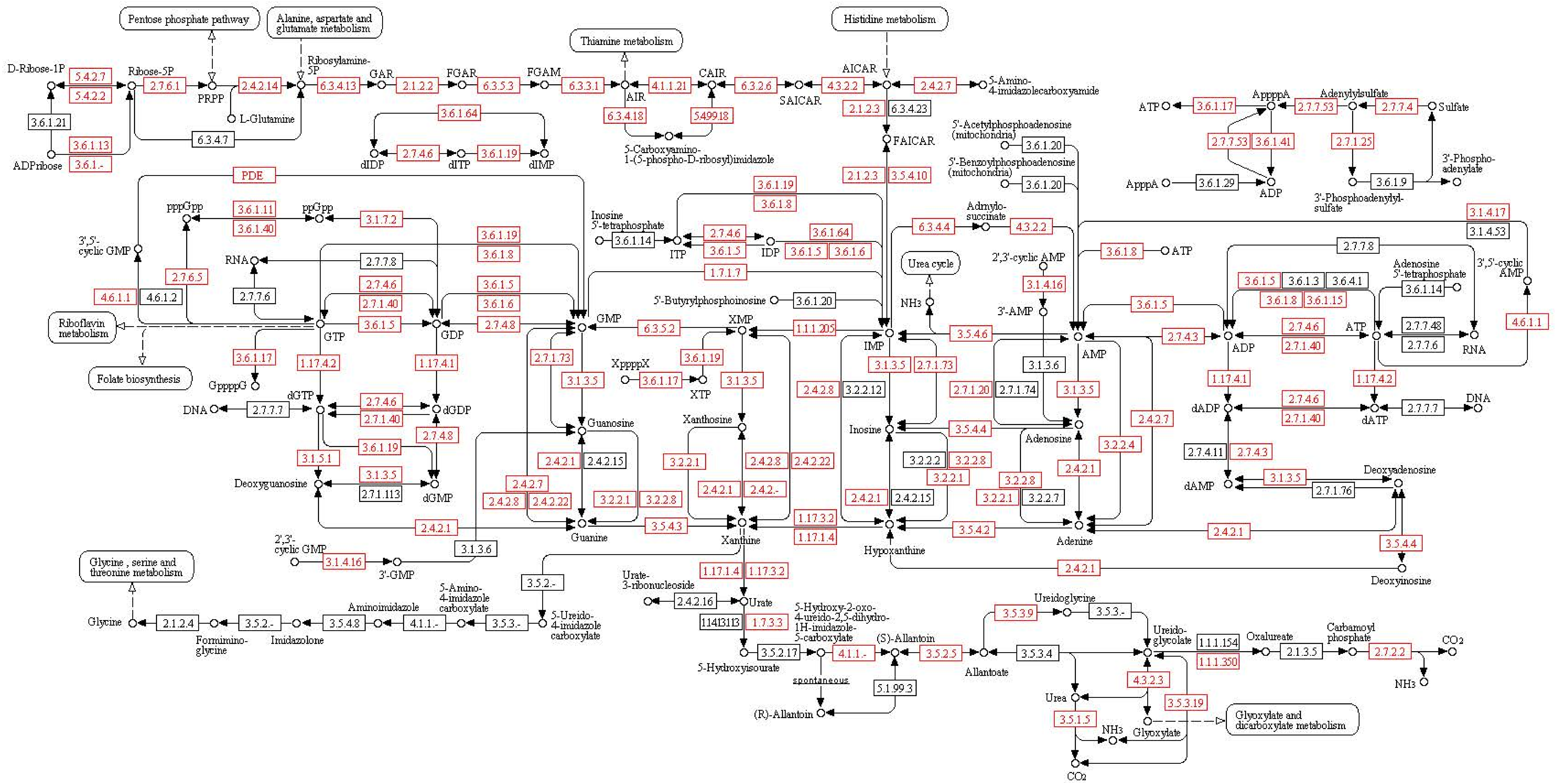

00230 11/7/13
(c) Kanehisa Laboratories

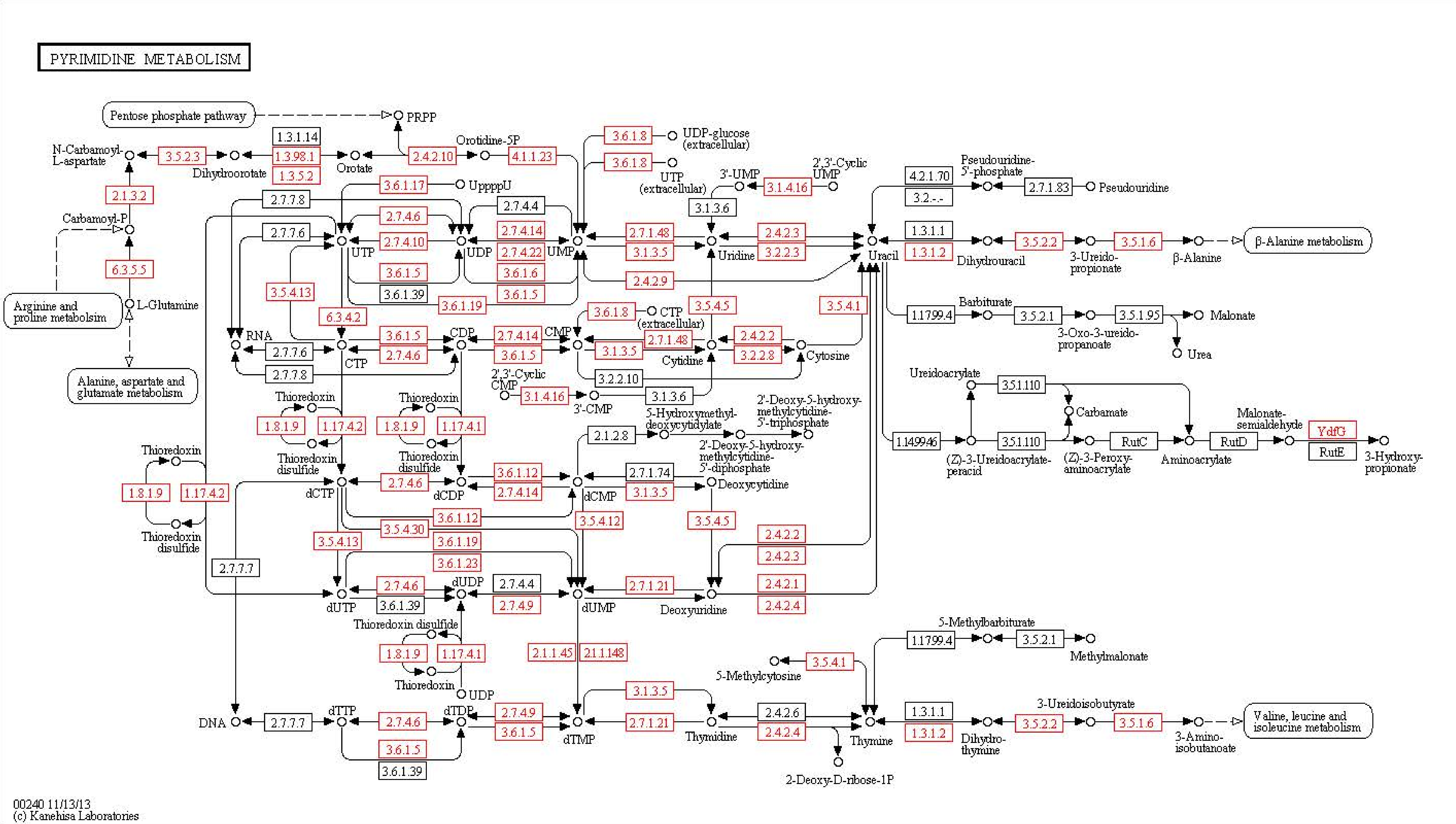
PYRIMIDINE METABOLISM
Pentose phosphate pathway
PRPP
N-Carbamoyl-L-aspartate
3.5.2.3
Dihydroorotate
1.3.1.14
1.3.98.1
1.3.5.2
Orotate
2.4.2.10
Orotidine-5P
4.1.1.23
2.1.3.2
Carbamoyl-P
6.3.5.5
Arginine and proline metabolism
L-Glutamine
Alanine, aspartate and glutamate metabolism
3.6.1.8
UDP-glucose (extracellular)
3.6.1.8
UTP (extracellular)
3.6.1.17
UppppU
2.7.7.8
2.7.4.4
2.7.4.6
2.7.4.14
2.7.7.6
2.7.4.10
UTP
UDP
2.7.4.22
UMP
3.6.1.5
3.6.1.6
3.6.1.39
3.6.1.19
3.6.1.5
3.5.4.13
6.3.4.2
RNA
2.7.7.6
CTP
3.6.1.5
CDP
2.7.4.6
2.7.4.14
3.6.1.5
CMP
2.7.7.8
3.6.1.8
CTP (extracellular)
2.7.1.48
3.1.3.5
Cytidine
3.2.2.10
2',3'-Cyclic CMP
3.1.4.16
3'-CMP
3.1.3.6
2.7.1.48
3.1.3.5
2.4.2.9
3'-UMP
3.1.4.16
2',3'-Cyclic UMP
3.1.3.6
Uridine
2.4.2.3
3.2.2.3
3.5.4.5
2.4.2.2
3.2.2.8
Cytosine
3.5.4.1
Uracil
4.2.1.70
3.2.-.-
Pseudouridine-5'-phosphate
2.7.1.83
Pseudouridine
1.3.1.1
1.3.1.2
Dihydrouracil
3.5.2.2
3-Ureido-propionate
3.5.1.6
β-Alanine
β-Alanine metabolism
1.17.99.4
Barbiturate
3.5.2.1
3-Oxo-3-ureido-propanoate
3.5.1.95
Malonate
Urea
Ureidoacrylate
3.5.1.110
Carbamate
1.14.99.46
(Z)-3-Ureidoacrylate-peracid
3.5.1.110
(Z)-3-Peroxy-aminoacrylate
RutC
Aminoacrylate
RutD
Malonate-semialdehyde
YdfG
RutE
3-Hydroxy-propionate
Thioredoxin
1.8.1.9
1.17.4.2
Thioredoxin disulfide
Thioredoxin
1.8.1.9
1.17.4.1
Thioredoxin disulfide
Thioredoxin
1.8.1.9
1.17.4.2
Thioredoxin disulfide
2.1.2.8
5-Hydroxymethyl-deoxycytidylate
2'-Deoxy-5-hydroxy-methylcytidine-5'-triphosphate
2'-Deoxy-5-hydroxy-methylcytidine-5'-diphosphate
dCTP
2.7.4.6
dCDP
3.6.1.12
2.7.4.14
dCMP
2.7.1.74
3.1.3.5
Deoxycytidine
3.6.1.12
3.5.4.30
3.5.4.13
3.6.1.19
3.6.1.23
3.5.4.12
3.5.4.5
2.4.2.2
2.4.2.3
2.4.2.1
2.4.2.4
2.7.7.7
dUTP
2.7.4.6
3.6.1.39
dUDP
2.7.4.4
2.7.4.9
dUMP
2.7.1.21
Deoxyuridine
Thioredoxin disulfide
1.8.1.9
1.17.4.1
Thioredoxin
UDP
2.1.1.45
2.1.1.148
5-Methylbarbiturate
1.17.99.4
3.5.2.1
Methylmalonate
5-Methylcytosine
3.5.4.1
DNA
2.7.7.7
dTTP
2.7.4.6
dTDP
2.7.4.9
3.6.1.5
3.6.1.5
3.6.1.39
dTMP
3.1.3.5
2.7.1.21
Thymidine
2.4.2.6
2.4.2.4
Thymine
2-Deoxy-D-ribose-1P
1.3.1.1
1.3.1.2
Dihydro-thymine
3-Ureidoisobutyrate
3.5.2.2
3.5.1.6
3-Amino-isobutanoate
Valine, leucine and isoleucine metabolism
00240 11/13/13
(c) Kanehisa Laboratories

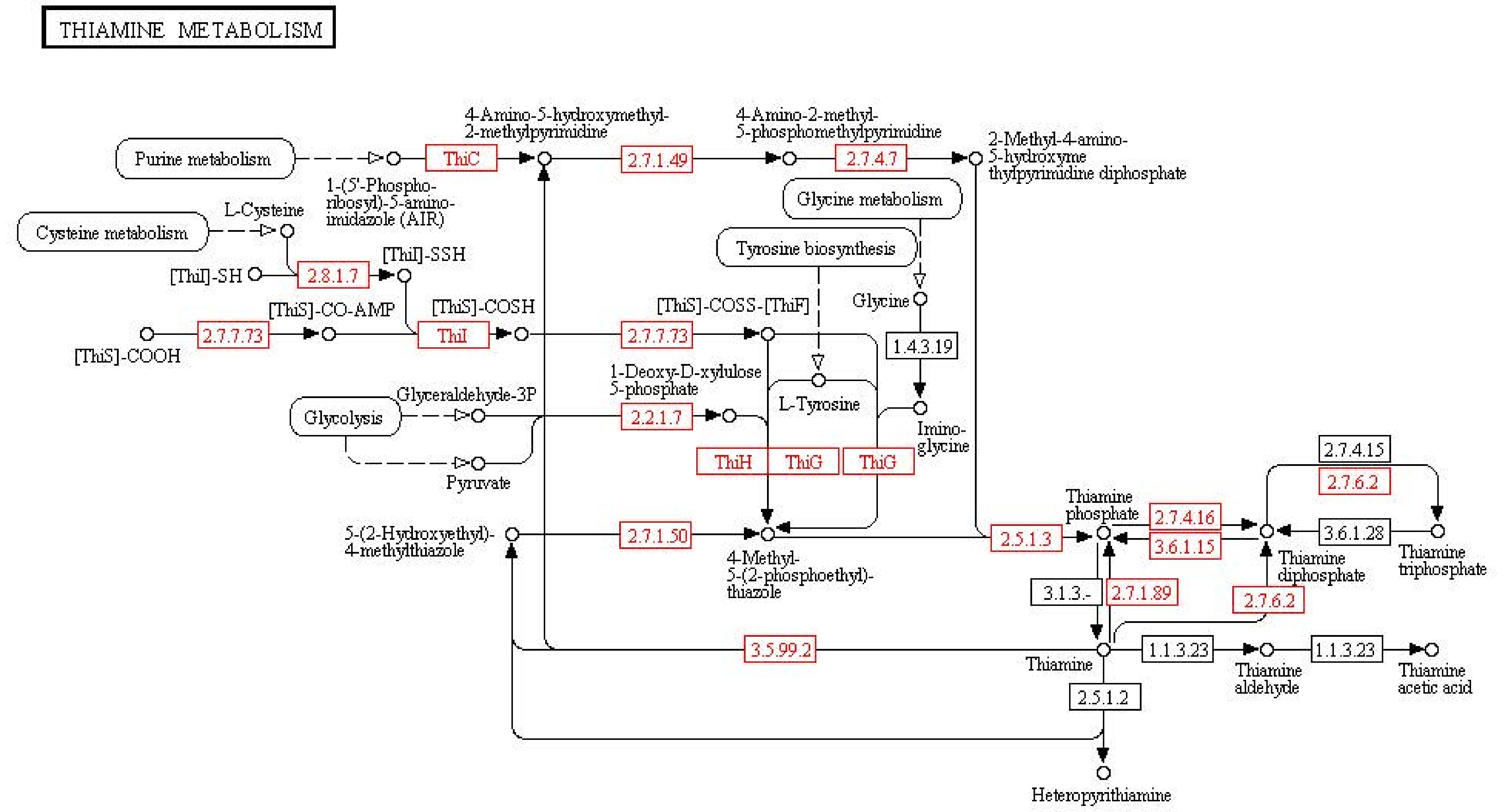

00730 4/26/13

(c) Kanehisa Laboratories

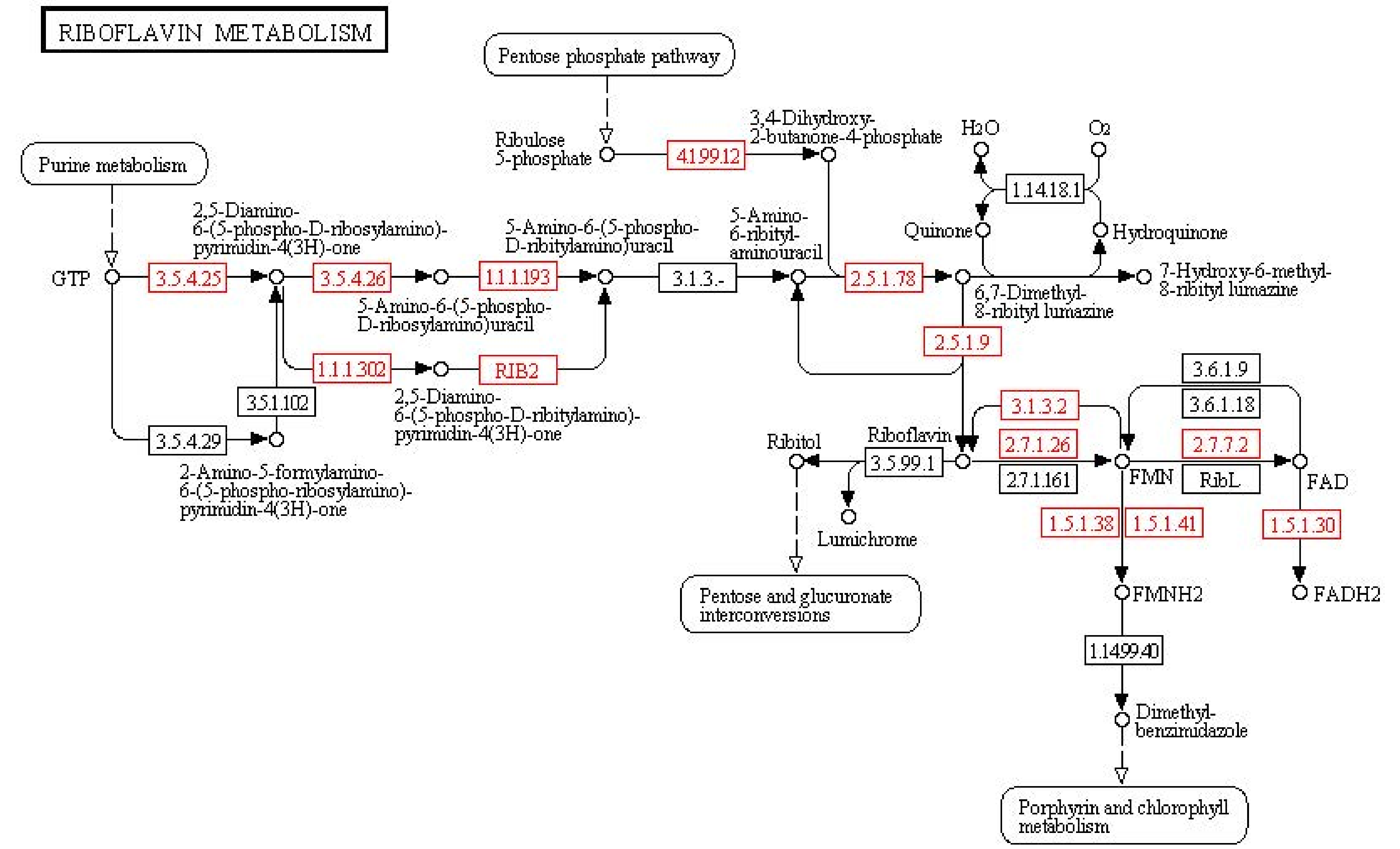

00740 6/8/12
(c) Kanehisa Laboratories

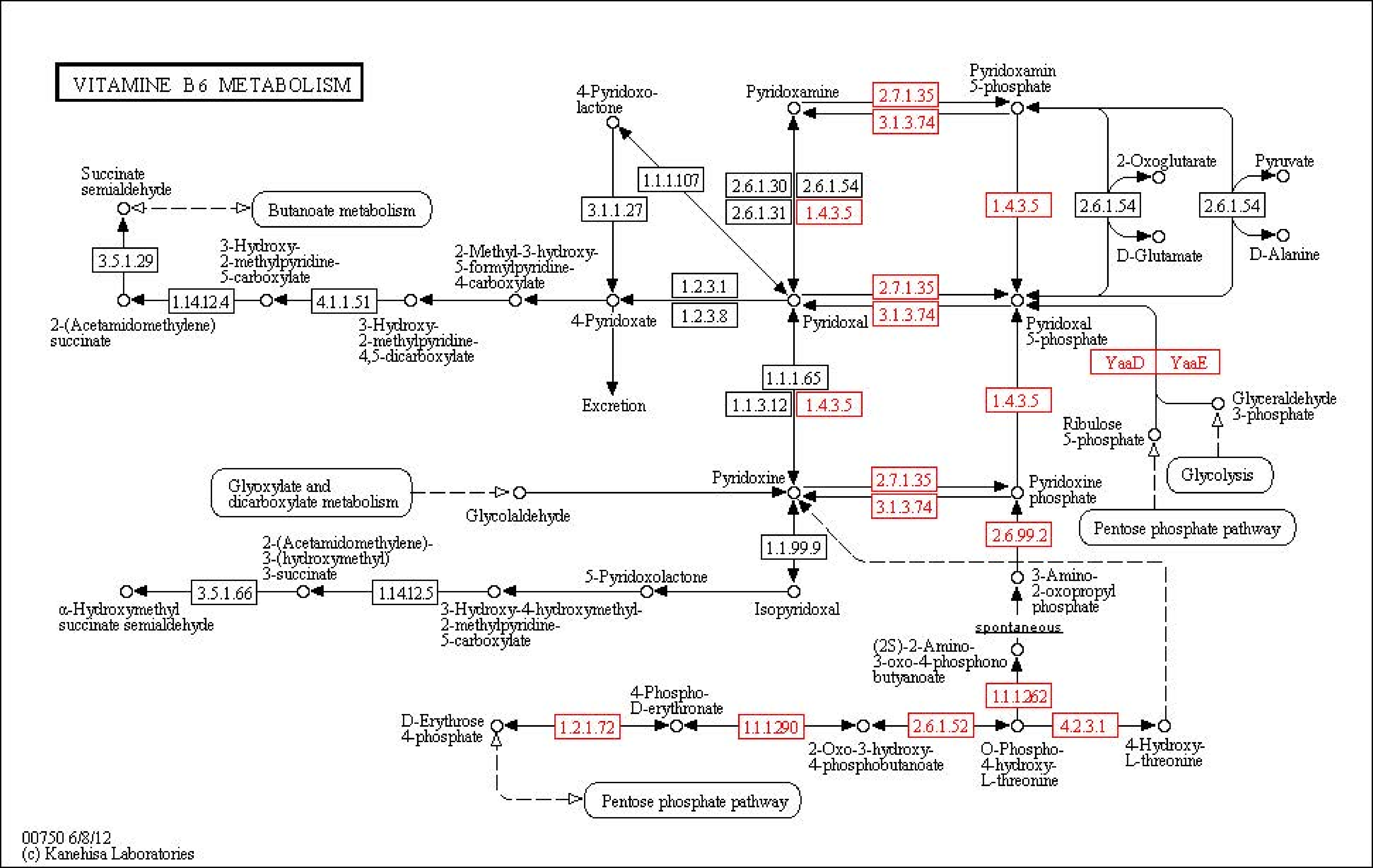

VITAMINE B6 METABOLISM
4-Pyridoxo-lactone
Pyridoxamine
Pyridoxamin 5-phosphate
2.7.1.35
3.1.3.74
Succinate semialdehyde
Butanoate metabolism
1.1.1.107
3.1.1.27
2.6.1.30
2.6.1.54
2.6.1.31
1.4.3.5
2-Oxoglutarate
Pyruvate
D-Glutamate
D-Alanine
3.5.1.29
3-Hydroxy-2-methylpyridine-5-carboxylate
2-Methyl-3-hydroxy-5-formylpyridine-4-carboxylate
1.2.3.1
1.2.3.8
1.14.12.4
4.1.1.51
2-(Acetamidomethylene) succinate
3-Hydroxy-2-methylpyridine-4,5-dicarboxylate
4-Pyridoxate
Pyridoxal
Pyridoxal 5-phosphate
YaaD
YaaE
1.1.1.65
1.1.3.12
Excretion
Glyceraldehyde 3-phosphate
Ribulose 5-phosphate
Glycolysis
Glyoxylate and dicarboxylate metabolism
Pyridoxine
Glycolaldehyde
Pyridoxine phosphate
Pentose phosphate pathway
2.6.99.2
1.1.99.9
2-(Acetamidomethylene)-3-(hydroxymethyl)-3-succinate
5-Pyridoxolactone
3-Amino-2-oxopropyl phosphate
3.5.1.66
1.14.12.5
α-Hydroxymethyl succinate semialdehyde
3-Hydroxy-4-hydroxymethyl-2-methylpyridine-5-carboxylate
Isopyridoxal
spontaneous
(2S)-2-Amino-3-oxo-4-phosphono butyanoate
1.1.1.262
4-Phospho-D-erythronate
D-Erythrose 4-phosphate
1.2.1.72
1.1.1.290
2.6.1.52
4.2.3.1
2-Oxo-3-hydroxy-4-phosphobutanoate
O-Phospho-4-hydroxy-L-threonine
4-Hydroxy-L-threonine
Pentose phosphate pathway
00750 6/8/12
(c) Kanehisa Laboratories

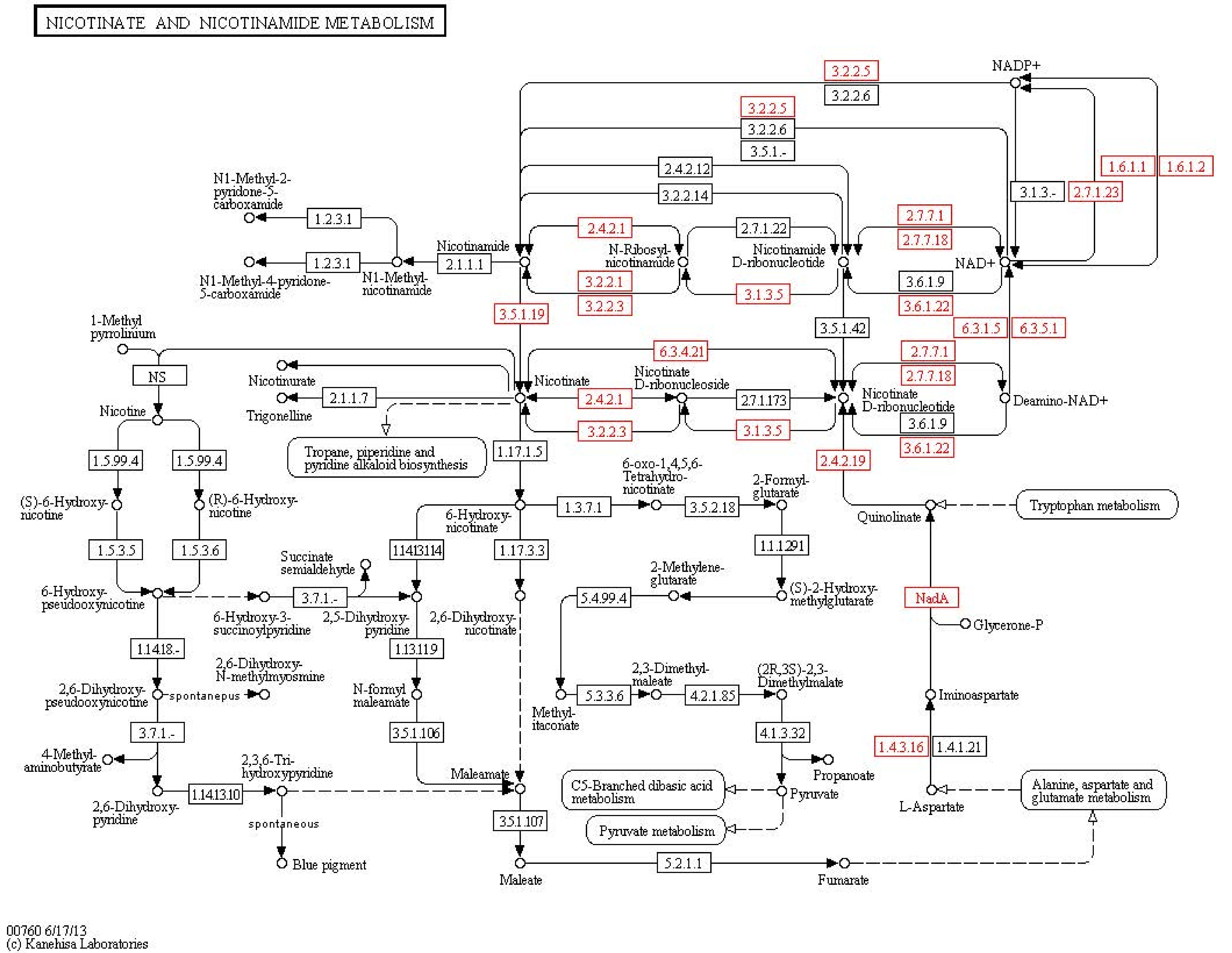
NICOTINATE AND NICOTINAMIDE METABOLISM
NADP+
3.2.2.5
3.2.2.6
3.2.2.5
3.2.2.6
3.5.1.-
2.4.2.12
3.2.2.14
1.6.1.1
1.6.1.2
3.1.3.-
2.7.1.23
N1-Methyl-2-pyridone-5-carboxamide
1.2.3.1
1.2.3.1
N1-Methyl-4-pyridone-5-carboxamide
N1-Methyl-nicotinamide
2.1.1.1
Nicotinamide
2.4.2.1
N-Ribosyl-nicotinamide
2.7.1.22
Nicotinamide D-ribonucleotide
2.7.7.1
2.7.7.18
NAD+
3.2.2.1
3.2.2.3
3.1.3.5
3.6.1.9
3.6.1.22
3.5.1.19
3.5.1.42
6.3.1.5
6.3.5.1
1-Methyl pyrrolinium
NS
Nicotine
Nicotinurate
Trigonelline
2.1.1.7
Nicotinate
6.3.4.21
Nicotinate D-ribonucleoside
2.4.2.1
2.7.1.173
Nicotinate D-ribonucleotide
2.7.7.1
2.7.7.18
Deamino-NAD+
3.2.2.3
3.1.3.5
3.6.1.9
3.6.1.22
2.4.2.19
Tropane, piperidine and pyridine alkaloid biosynthesis
1.17.1.5
1.5.99.4
1.5.99.4
(S)-6-Hydroxy-nicotine
(R)-6-Hydroxy-nicotine
6-oxo-1,4,5,6-Tetrahydro-nicotinate
2-Formyl-glutarate
1.3.7.1
3.5.2.18
6-Hydroxy-nicotinate
Quinolinate
Tryptophan metabolism
1.5.3.5
1.5.3.6
1.14.13.114
1.17.3.3
1.1.1.291
Succinate semialdehyde
2-Methylene-glutarate
(S)-2-Hydroxy-methylglutarate
6-Hydroxy-pseudooxynicotine
3.7.1.-
5.4.99.4
NadA
6-Hydroxy-3-succinoylpyridine
2,5-Dihydroxy-pyridine
2,6-Dihydroxy-nicotinate
Glycerone-P
1.14.18.-
1.13.11.9
2,6-Dihydroxy-N-methylmyosmine
2,3-Dimethyl-maleate
(2R,3S)-2,3-Dimethylmalate
2,6-Dihydroxy-pseudooxynicotine
spontanepus
N-formyl maleamate
5.3.3.6
4.2.1.85
Iminoaspartate
Methyl-itaconate
3.7.1.-
3.5.1.106
4.1.3.32
1.4.3.16
1.4.1.21
4-Methyl-aminobutyrate
2,3,6-Tri-hydroxypyridine
Maleamate
Propanoate
C5-Branched dibasic acid metabolism
Pyruvate
Alanine, aspartate and glutamate metabolism
L-Aspartate
2,6-Dihydroxy-pyridine
1.14.13.10
spontaneous
3.5.1.107
Pyruvate metabolism
Blue pigment
Maleate
5.2.1.1
Fumarate
00760 6/17/13
(c) Kanehisa Laboratories

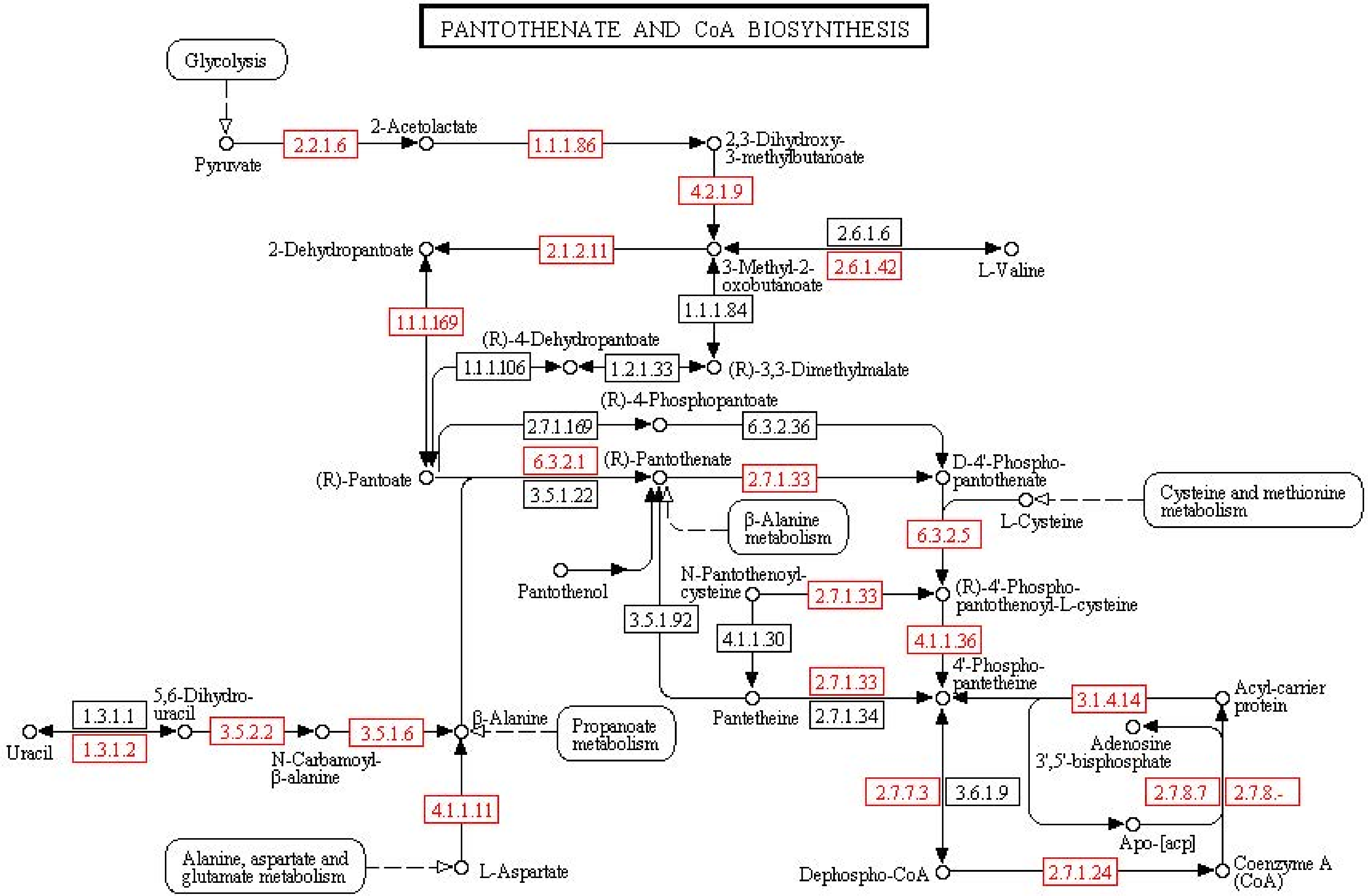

00770 6/8/12
(c) Kanehisa Laboratories

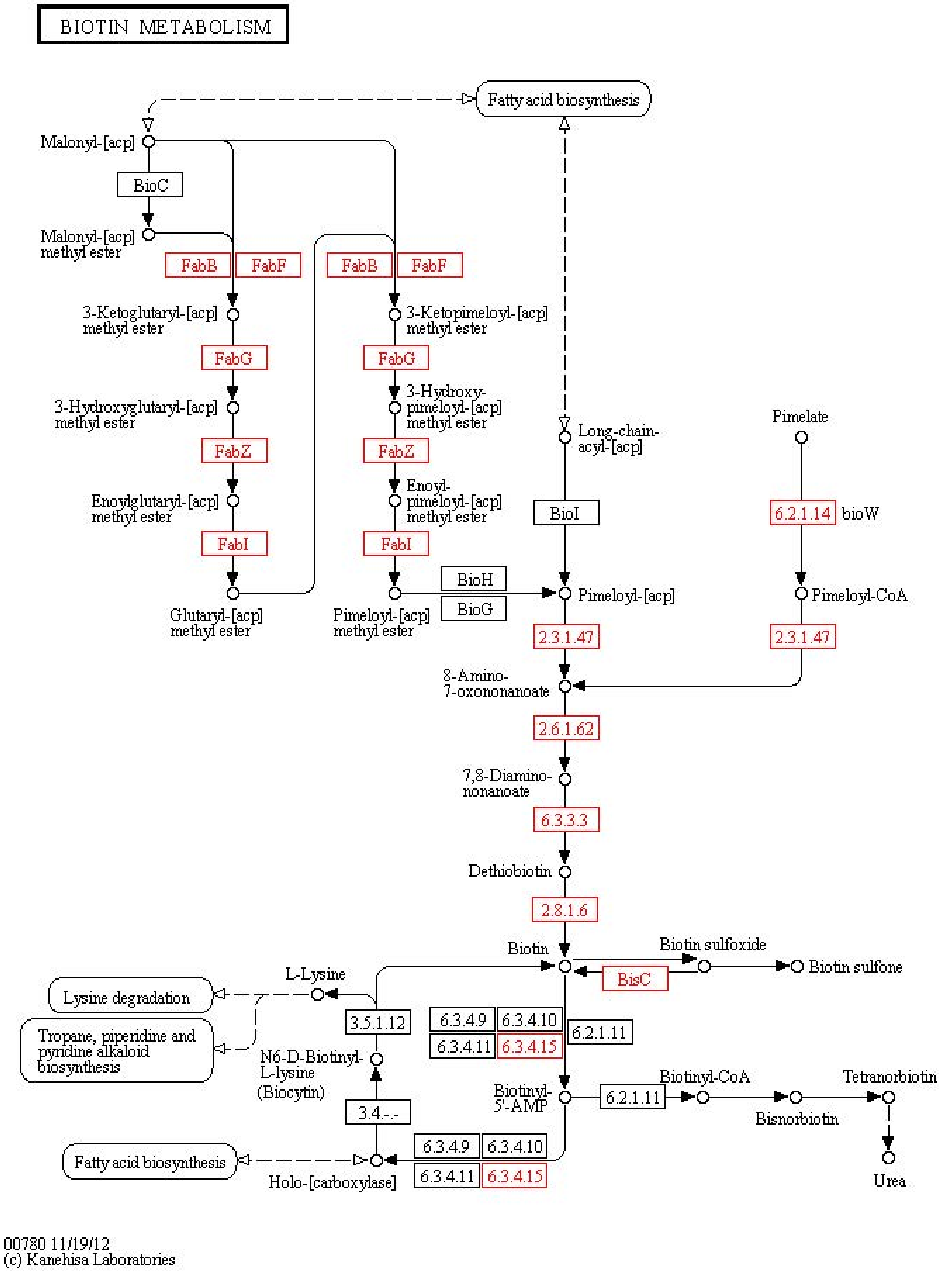
BIOTIN METABOLISM
Fatty acid biosynthesis
Malonyl-[acp]
BioC
Malonyl-[acp] methyl ester
FabB
FabF
FabB
FabF
3-Ketoglutaryl-[acp] methyl ester
3-Ketopimeloyl-[acp] methyl ester
FabG
FabG
3-Hydroxyglutaryl-[acp] methyl ester
3-Hydroxy-pimeloyl-[acp] methyl ester
FabZ
FabZ
Enoylglutaryl-[acp] methyl ester
Enoyl-pimeloyl-[acp] methyl ester
FabI
FabI
Glutaryl-[acp] methyl ester
Pimeloyl-[acp] methyl ester
BioH
BioG
Long-chain-acyl-[acp]
BioI
Pimelate
6.2.1.14
bioW
Pimeloyl-[acp]
Pimeloyl-CoA
2.3.1.47
2.3.1.47
8-Amino-7-oxononanoate
2.6.1.62
7,8-Diamino-nonanoate
6.3.3.3
Dethiobiotin
2.8.1.6
Biotin
Biotin sulfoxide
Biotin sulfone
BisC
L-Lysine
Lysine degradation
Tropane, piperidine and pyridine alkaloid biosynthesis
3.5.1.12
6.3.4.9
6.3.4.10
6.3.4.11
6.3.4.15
6.2.1.11
N6-D-Biotinyl-L-lysine (Biocytin)
3.4.-.-
Biotinyl-5'-AMP
6.2.1.11
Biotinyl-CoA
Bisnorbiotin
Tetranorbiotin
Urea
6.3.4.9
6.3.4.10
6.3.4.11
6.3.4.15
Fatty acid biosynthesis
Holo-[carboxylase]
00780 11/19/12
(c) Kanehisa Laboratories

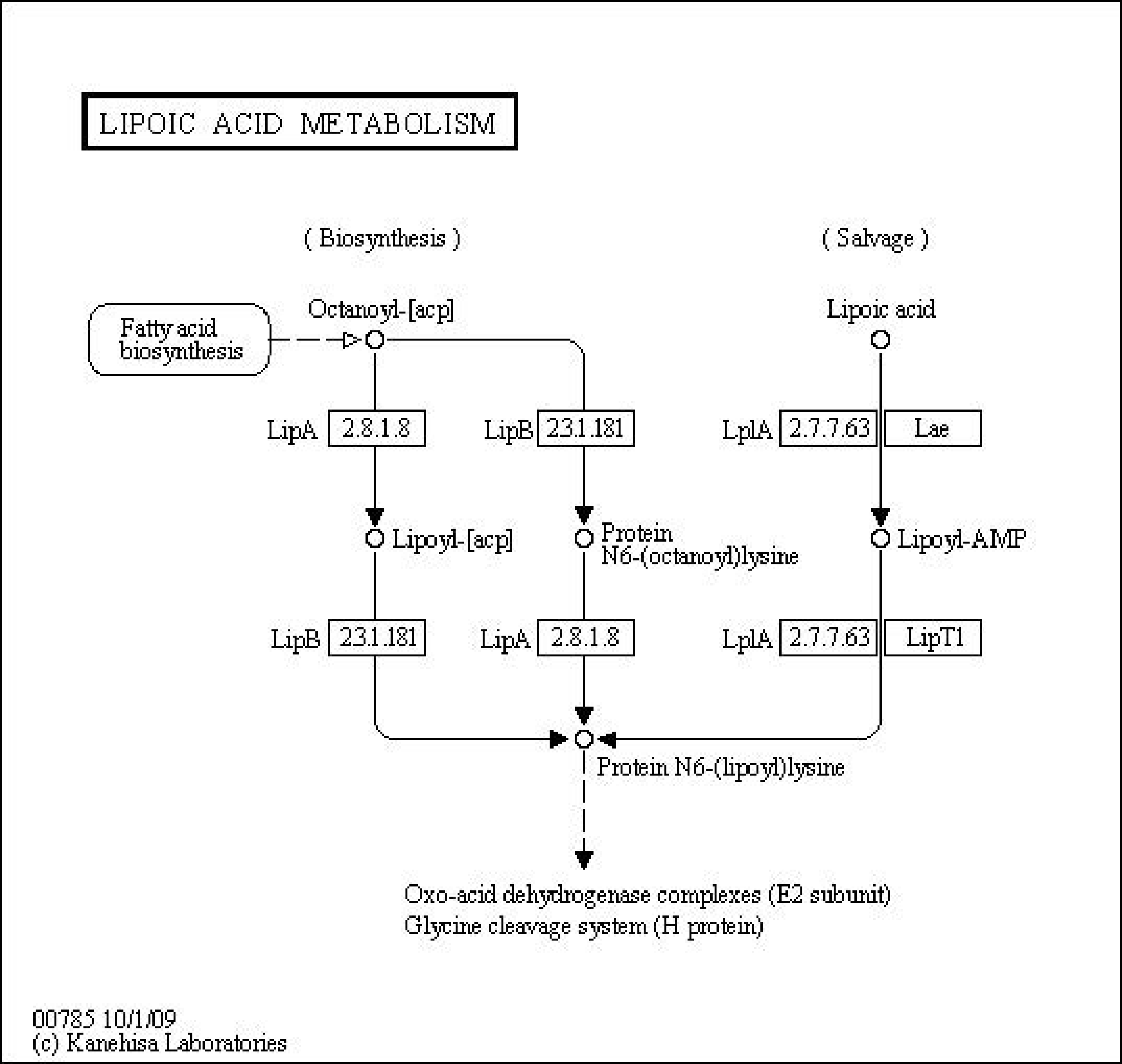
LIPOIC ACID METABOLISM
(Biosynthesis)
(Salvage)
Fatty acid biosynthesis
Octanoyl-[acp]
Lipoic acid
LipA
2.8.1.8
LipB
2.3.1.181
LplA
2.7.7.63
Lae
Lipoyl-[acp]
Protein N6-(octanoyl)lysine
Lipoyl-AMP
LipB
2.3.1.181
LipA
2.8.1.8
LplA
2.7.7.63
LipT1
Protein N6-(lipoyl)lysine
Oxo-acid dehydrogenase complexes (E2 subunit)
Glycine cleavage system (H protein)
00785 10/1/09
(c) Kanehisa Laboratories

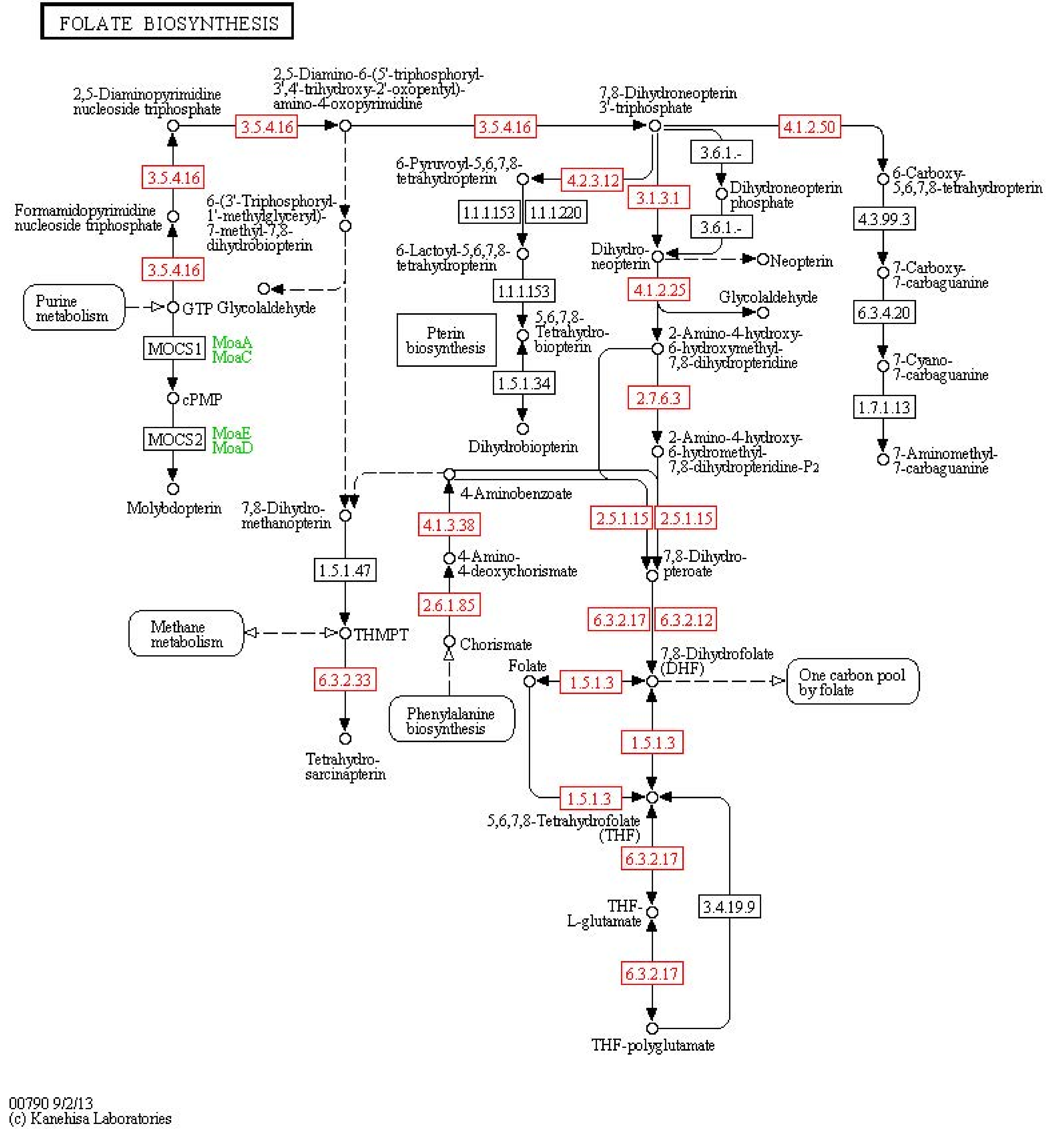

FOLATE BIOSYNTHESIS
2,5-Diaminopyrimidine nucleoside triphosphate
2,5-Diamino-6-(5'-triphosphoryl-3',4'-trihydroxy-2'-oxopentyl)-amino-4-oxopyrimidine
7,8-Dihydroneopterin 3'-triphosphate
3.5.4.16
3.5.4.16
4.1.2.50
3.6.1.-
6-Pyruvoyl-5,6,7,8-tetrahydropterin
4.2.3.12
3.1.3.1
Dihydroneopterin phosphate
6-Carboxy-5,6,7,8-tetrahydropterin
3.5.4.16
Formamidopyrimidine nucleoside triphosphate
6-(3'-Triphosphoryl-1'-methylglyceryl)-7-methyl-7,8-dihydrobiopterin
1.1.1.153
1.1.1.220
3.6.1.-
4.3.99.3
6-Lactoyl-5,6,7,8-tetrahydropterin
Dihydro-neopterin
Neopterin
7-Carboxy-7-carbaguanine
3.5.4.16
1.1.1.153
4.1.2.25
Glycolaldehyde
Purine metabolism
GTP Glycolaldehyde
5,6,7,8-Tetrahydro-biopterin
6.3.4.20
Pterin biosynthesis
2-Amino-4-hydroxy-6-hydroxymethyl-7,8-dihydropteridine
MOCS1
MoaA
MoaC
7-Cyano-7-carbaguanine
1.5.1.34
cPMP
2.7.6.3
1.7.1.13
MOCS2
MoaE
MoaD
Dihydrobiopterin
2-Amino-4-hydroxy-6-hydromethyl-7,8-dihydropteridine-P2
7-Aminomethyl-7-carbaguanine
4-Aminobenzoate
Molybdopterin
7,8-Dihydro-methanopterin
4.1.3.38
2.5.1.15
2.5.1.15
4-Amino-4-deoxychorismate
7,8-Dihydro-pteroate
1.5.1.47
2.6.1.85
6.3.2.17
6.3.2.12
Methane metabolism
THMPT
Chorismate
7,8-Dihydrofolate (DHF)
Folate
1.5.1.3
One carbon pool by folate
6.3.2.33
Phenylalanine biosynthesis
1.5.1.3
Tetrahydro-sarcinapterin
1.5.1.3
5,6,7,8-Tetrahydrofolate (THF)
6.3.2.17
THF-L-glutamate
3.4.19.9
6.3.2.17
THF-polyglutamate
00790 9/2/13
(c) Kanehisa Laboratories

ONE CARBON POOL BY FOLATE

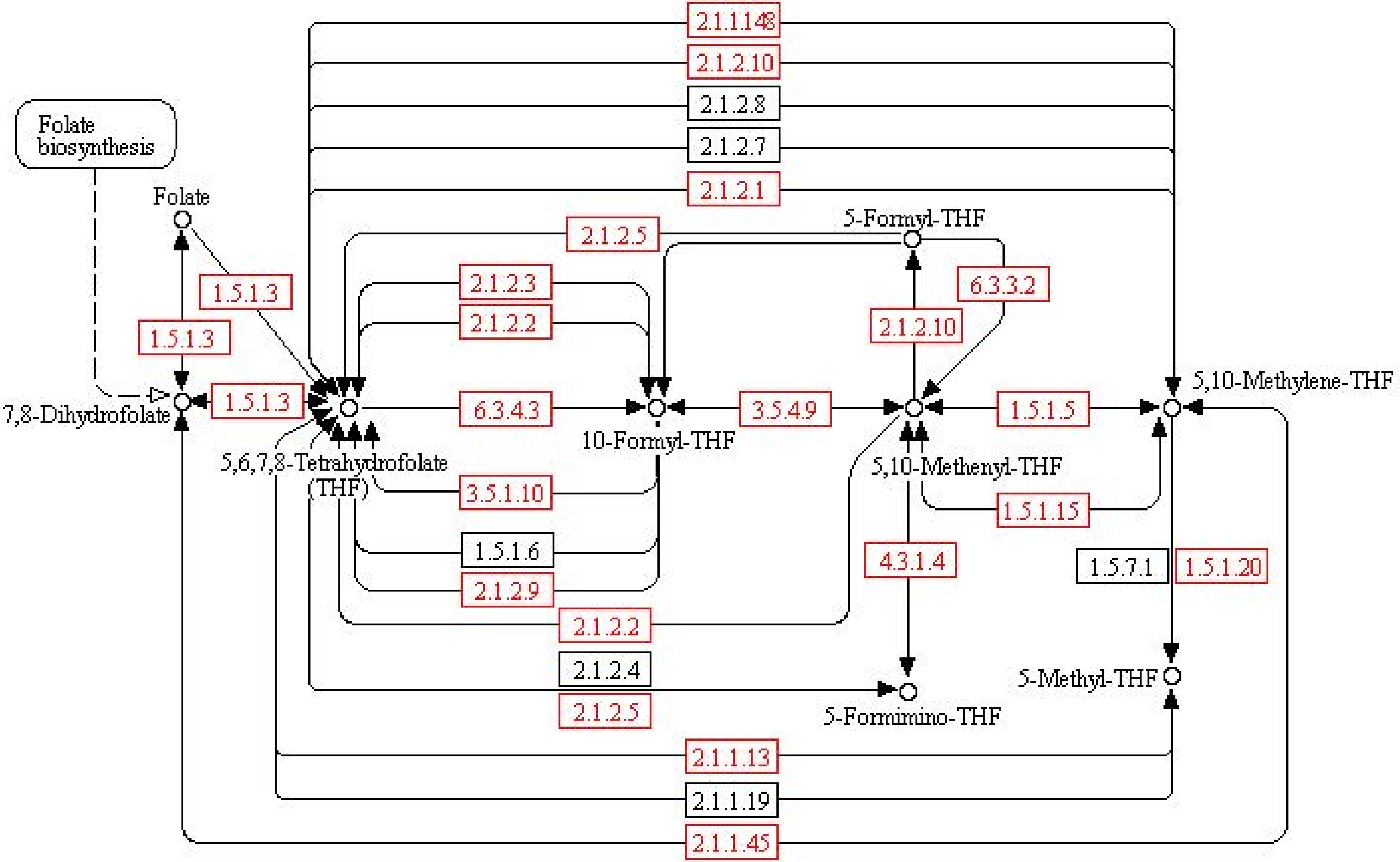

00670 9/13/11
(c) Kanehisa Laboratories

PORPHYRIN AND CHLOROPHYLL METABOLISM

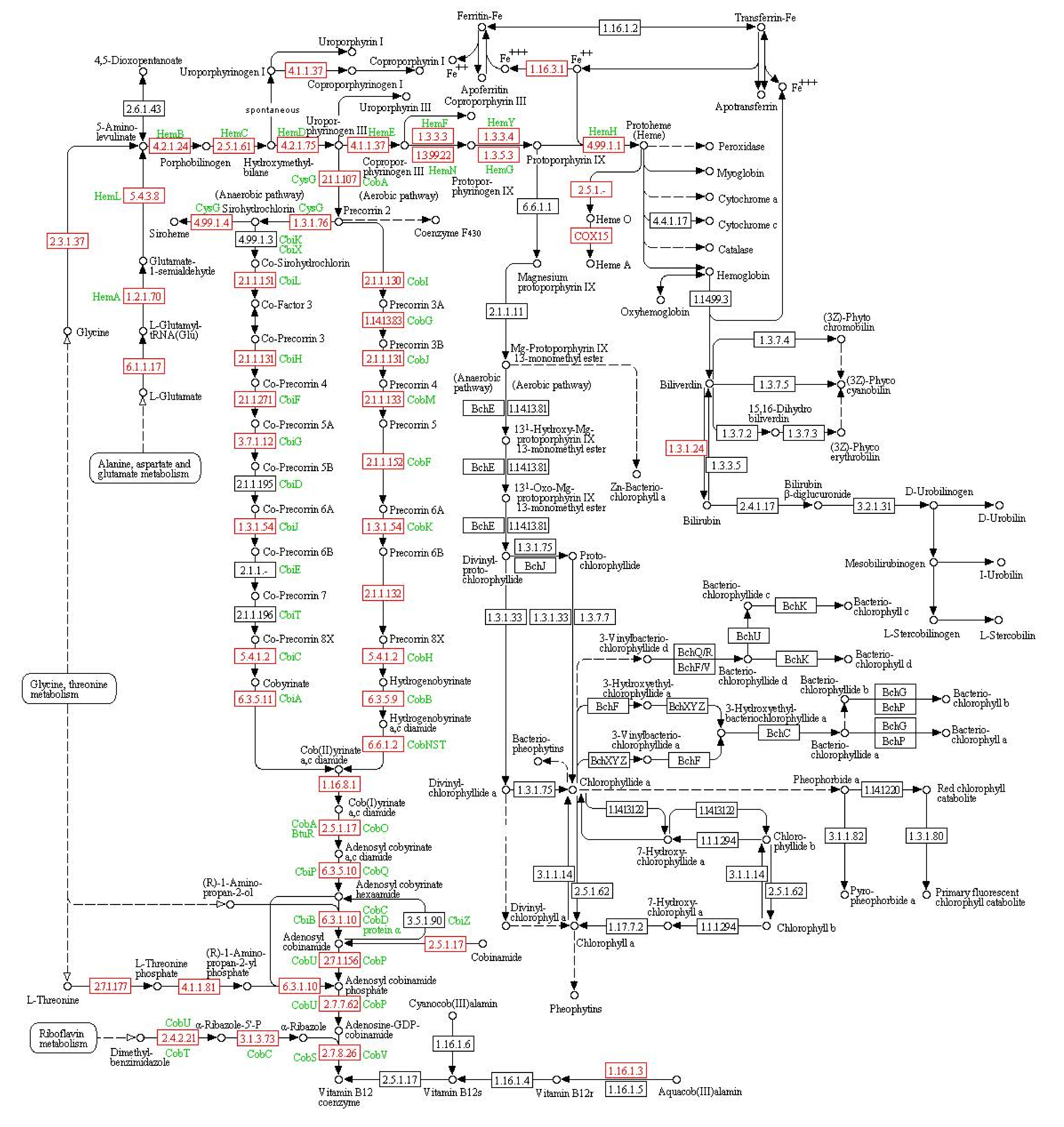

00860 7/29/13
(c) Kanehisa Laboratories

UBIQUINONE AND OTHER TERPENOID-QUINONE BIOSYNTHESIS

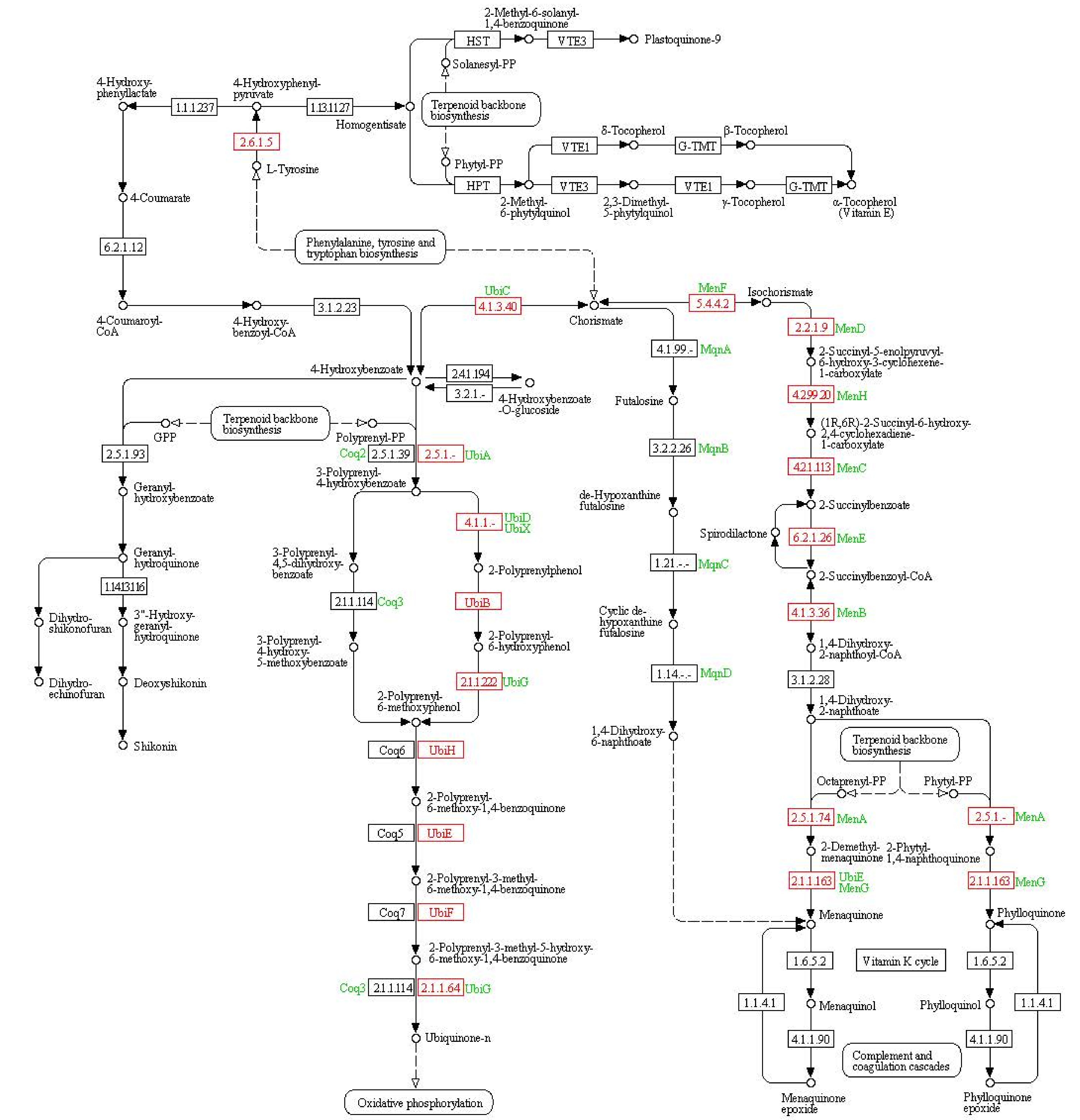

00130 6/19/12
(c) Kanehisa Laboratories
