# Supplementary figures and images for: Genome-scale metabolic reconstructions of Bifidobacterium adolescentis L2-32 and Faecalibacterium prausnitzii A2-165 and their interaction
Source: BMC Syst Biol. 2014 Apr 3;8:41. doi: 10.1186/1752-0509-8-41 (PMC4108055; doi:10.1186/1752-0509-8-41)

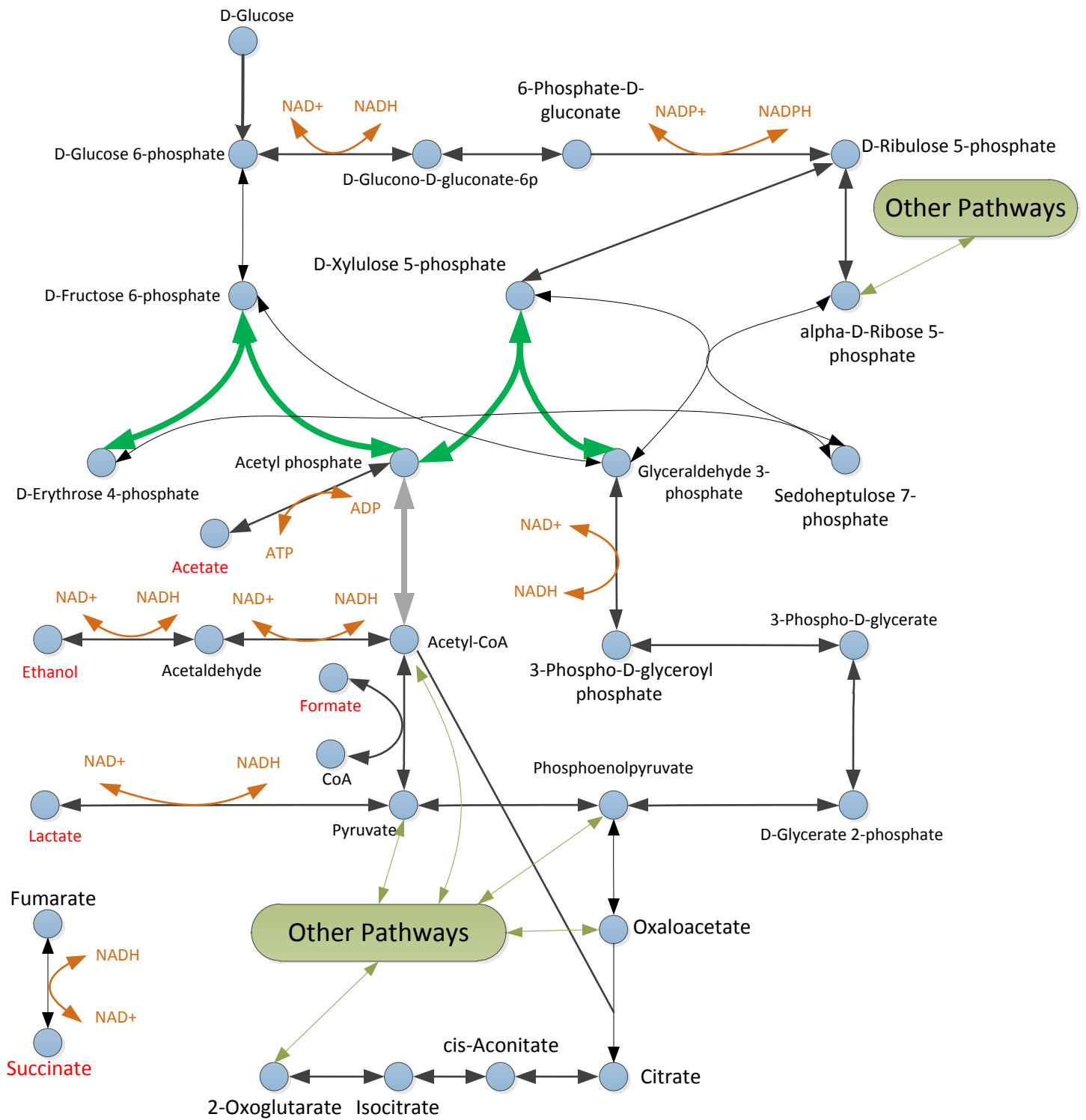

Supplement: Additional file 9: Figure S3 — Main carbon metabolism in Bifidobacterium adolescentis adolescentis L2-32. [file 1752-0509-8-41-S9.pdf]

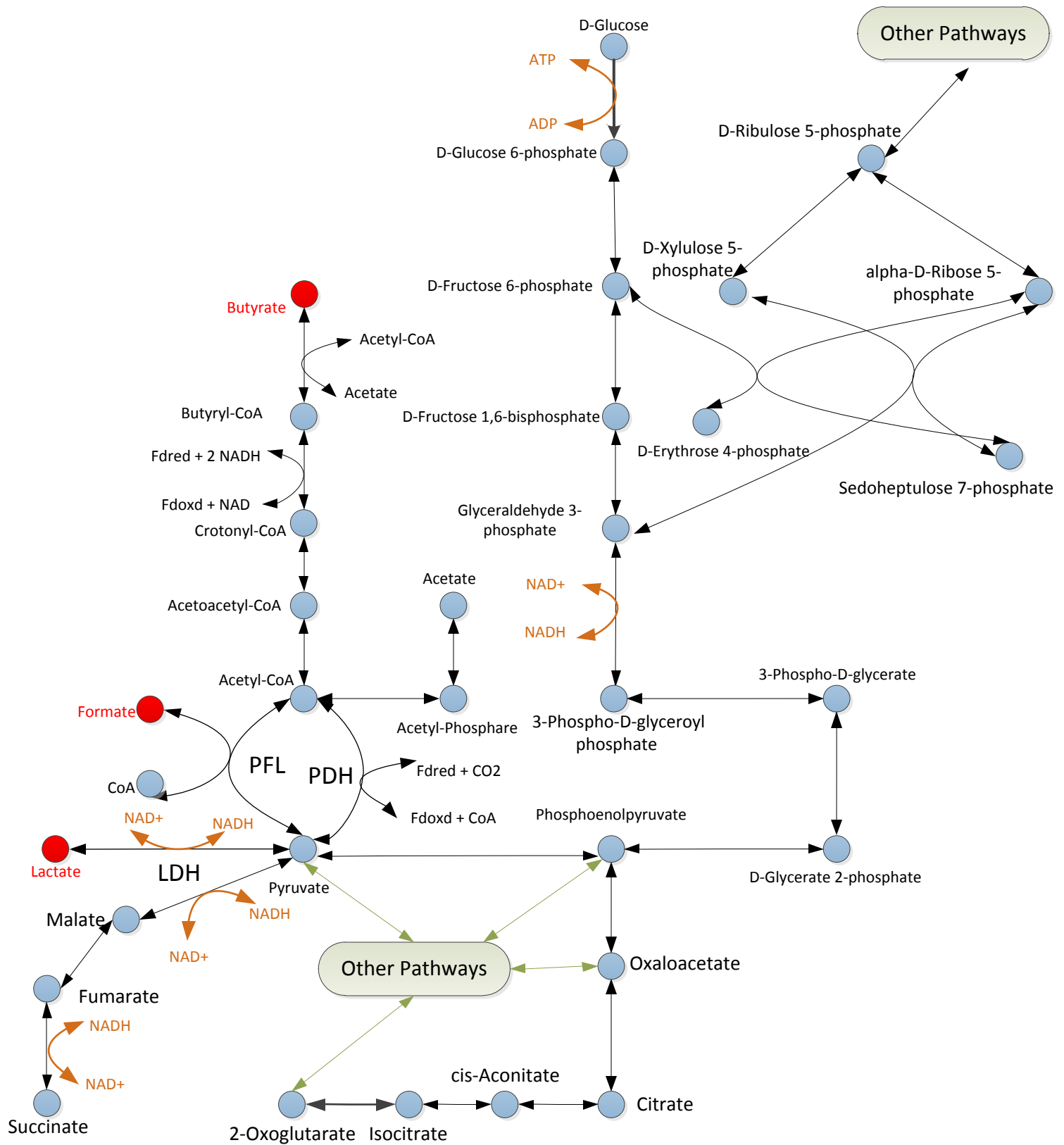

Supplement: Additional file 10: Figure S4 — Main carbon metabolism in Faecalibacterium prausnitzii A2-165. [file 1752-0509-8-41-S10.pdf]
